# *Blattella germanica* displays a large arsenal of antimicrobial peptide genes

**DOI:** 10.1038/s41598-020-77982-3

**Published:** 2020-12-03

**Authors:** Francisco J. Silva, Maria Muñoz-Benavent, Carlos García-Ferris, Amparo Latorre

**Affiliations:** 1 Institute for Integrative Systems Biology (I2SysBio), University of Valencia-CSIC, Paterna, Spain; 2 Genomics and Health Area, Foundation for the Promotion of Sanitary and Biomedical Research, Valencia, Spain; 3 Department of Biochemistry and Molecular Biology, University of Valencia, Valencia, Spain

**Keywords:** Genome evolution, Evolutionary biology, Antimicrobial responses, Entomology, Symbiosis

## Abstract

Defence systems against microbial pathogens are present in most living beings. The German cockroach *Blattella germanica* requires these systems to adapt to unhealthy environments with abundance of pathogenic microbes, in addition to potentially control its symbiotic systems. To handle this situation, four antimicrobial gene families (defensins, termicins, drosomycins and attacins) were expanded in its genome. Remarkably, a new gene family (blattellicins) emerged recently after duplication and fast evolution of an attacin gene, which is now encoding larger proteins with the presence of a long stretch of glutamines and glutamic acids. Phylogenetic reconstruction, within Blattellinae, suggests that this duplication took place before the divergence of *Blattella* and *Episymploce* genera. The latter harbours a long attacin gene (pre-blattellicin), but the absence of the encoded Glx-region suggests that this element evolved recently in the *Blattella* lineage. A screening of AMP gene expression in available transcriptomic SR projects of *B. germanica* showed that, while some AMPs are expressed during almost the whole development, others are restricted to shorter periods. Blattellicins are highly expressed only in adult females. None of the available SR tissue projects could be associated with blattellicins’ expression, suggesting that it takes place in other tissues, maybe the gut.

## Introduction

Multicellular eukaryotic genomes encode large sets of genes involved in defence mechanisms against microbial pathogens. The innate immune system controls the production of a variety of different types of proteins named antimicrobial peptides (AMPs), found in both vertebrate and invertebrate species. In insects, after the description of the first AMP *in Hyalophora cecropia* ^1^, hundreds were reported and are available in protein databases^2,3^. The German cockroach *Blattella germanica* (Order Blattodea) is a global pest in human settings including hospitals and schools. It is a vector of disease agents due to its lifestyle in unhealthy habitats. To defend against microbial pathogens, *B. germanica*, as other cockroaches, contains genes encoding AMPs. The sequencing of the cockroach genomes of *Periplaneta americana*^4^ and *B. germanica*^5,6^ revealed the presence of at least several types of AMP genes, encoding Attacins, Defensins, Drosomycins, Pro-rich peptides and Termicins.

AMPs are small proteins, usually below 100 residues^3^. Most are generally cationic and amphipathic, but there are more than 100 examples of naturally occurring anionic peptides with antimicrobial potential^7^. This implies that electrostatic interactions alone cannot explain their mode of action^8^. AMPs and lysozymes are among the main effector molecules that insects use to defend against those pathogens that arrive to the midgut or get inside the body. They are included among the type of proteins that induce pathogen death via lysis^9^. However, in contrast to lysozymes, AMPs synthesis used to be induced by several mechanisms such as the Toll and Imd signalling pathways. In insects, they are produced at diverse epithelial surfaces or at internal tissues such as fat body cells, to be released to the haemolymph and distributed to the whole body. The classically considered mode of action was their binding to microbial membranes, followed by the production of pores, membrane permeabilization and cell lysis. However, diverse types of actions on specific targets such as inhibition of protein synthesis or of bacterial cytokinesis have also been reported^10,11^.

The characterization of genes repertoires in plant and animal genomes revealed that most organisms harbour from five to ten distinct AMP gene families, frequently composed by several paralogous genes^11^. The evolutionary histories of AMP gene families during insect evolution were very dynamic with frequent duplications, deletions and pseudogenization events, but also de novo gene formation^11,12^. Initially, AMP proteins were considered to be not specific of concrete microbial species but to affect a broad range of organisms (Gram-negative bacteria, Gram-positive bacteria, fungi, etc.). However, recent studies based on *Drosophila* AMP gene inactivation revealed a complex pattern with some AMP gene products acting on reduced number of species, while others having a taxonomically broader effect^10^.

One additional function of AMPs is the regulation of endosymbiont or exosymbiont communities. In the weevil genus *Sitophilus*, one specific AMP, Coleoptericin A, evolved to control the mutualist endosymbiont *Candidatus* Sodalis pierantonius^13^ by preventing its escape from the bacteriocytes and its spread to other cells^14^. Permeabilization of symbiont membranes to solve the problem of the transport of metabolites between host cells and endosymbionts through sublethal AMP doses was also suggested^15^. *B. germanica* harbours specialized cells in the fat body, named bacteriocytes, which contain thousands of cells of the bacterial endosymbiont *Blattabacterium cuenoti* (hereafter, *Blattabacterium*). This symbiotic relationship is at least 150 My old and, with the exception of some endosymbiont losses, *Blattabacterium* and Blattodea hosts have been co-evolving since then^16–19^. The main role of the endosymbiont is to participate in host’s nitrogen economy^20,21^. Additionally, cockroaches are unique among insects as they also harbour a complex and rich microbiota in the hindgut, which composition and structure could be determined by the effect of specific AMPs^22–24^.

Antimicrobial peptides may serve as an alternative to solve the problem of antibiotic resistance in bacteria and have many biotechnological applications. In this work, we have carried out a thorough characterization of *B. germanica* AMPs describing 39 genes including an unusual Glx-rich new attacin-derived type that we have called blattellicin. The expression of each AMP gene may occur during the whole development or may be restricted to some stages, as it is the case of blattellicins.

## Results

### Genes encoding proteins with AMP domains in the genome of *B. germanica*

To identify annotated genes with AMP functions in the genome of *B. germanica*^6^ two strategies were used. The first was the search for product names including the terms defense, drosomycin, tenecin, phormicin, attacin and coleoptericin. The second was the search of annotated Pfam domains related to antimicrobial peptides. They are included in three clan domains of Pfam database: Knottin_1 (CL0054, Scorpion toxin-like knottin superfamily), Defensin (CL0075, Defensin/myotoxin-like superfamily) and Omega_toxin (CL0083, Omega toxin-like). The five detected Pfam domains were: PF11581 (Argos), PF03769 (Attacin_C), PF01097 (Defensin_2), PF00304 (Gamma-thionin) and PF11415 (Toxin_37). After the removal of C0J52_07645 (Giant-lens protein) and C0J52_08617 (putative defense protein 3), because they do not encode AMPs, 24 coding genes were retained (Supplementary Table 1). They were initially classified into the following groups: (i) Defensin_2 proteins (hereafter Defensin) (10 CDS, including two with the annotation partial = 5′), (ii) Drosomycin (Gamma-thionin domain) (10 CDS), (iii) Termicin (Toxin_37 domain) (3 CDS) and (iv) the CDS C0J52_26498. The latter, annotated as hypothetical protein, was a long protein (541 amino acids) with an Attacin_C domain. However, a less stringent domain analysis showed the potential presence of two or three additional domains in this protein with similarities with Attacin_C and Coleoptericin (PF06286).

**Table 1:** Genes encoding proteins with antimicrobial peptide domains in *B. germanica*.

No	Gene	Product	CDS L	Prot. L	PFAM code	PFAM name	Scaffold	Equivalent CDS
1	*defensin_g1*	Tenecin-1	225	74	PF01097	Defensin_2	Unplaced	
2	*defensin_g2*	Tenecin-1	225	74	PF01097	Defensin_2	PYGN01002380	C0J52_24001
3	*defensin_g3*	Tenecin-1	225	74	PF01097	Defensin_2	PYGN01003429	
4	*defensin_g4*	Tenecin-1	225	74	PF01097	Defensin_2	PYGN01003429	C0J52_27569
5	*defensin_g5*	Tenecin-1	225	74	PF01097	Defensin_2	PYGN01001185	C0J52_22338
6	*defensin_g6*	Tenecin-1	225	74	PF01097	Defensin_2	PYGN01002380	C0J52_24004
7	*defensin_g7*	Tenecin-1	225	74	PF01097	Defensin_2	PYGN01002380	
8	*defensin_g8*	Tenecin-1	225	74	PF01097	Defensin_2	PYGN01001185	C0J52_22336
9	*defensin_g9*	Phormicin	216	71	PF01097	Defensin_2	PYGN01002380	C0J52_24005
10	*defensin_g10*	Phormicin	216	71	PF01097	Defensin_2	PYGN01001185	
11	*defensin_g11*	Tenecin-1	246	81	PF01097	Defensin_2	PYGN01002380	C0J52_24006
12	*defensin_g12*	Tenecin-1	246	81	PF01097	Defensin_2	PYGN01001185	C0J52_22340
13	*defensin_g13*	Defense_Protein_6	228	75	PF01097	Defensin_2	PYGN01001185	C0J52_22339
14	*defensin_g14*	Defense_Protein_6	228	75	PF01097	Defensin_2	PYGN01001185	
15	*defensin_g15*	Defensin-like	192	63	PF01097	Defensin_2	PYGN01000358	C0J52_20459
16	*defensin_g16*	Defensin-like	192	63	PF01097	Defensin_2	PYGN01000358	C0J52_20460
17	*termicin_g1*	Termicin	195	64	PF11415	Toxin_37	PYGN01000196	C0J52_00758
18	*termicin_g2*	Termicin	195	64	PF11415	Toxin_37	PYGN01002934	C0J52_26761
19	*termicin_g3*	Termicin	195	64	PF11415	Toxin_37	PYGN01002934	C0J52_26762
20	*drosomycin_g1*	Drosomycin	201	66	PF00304	Gamma-thionin	Unplaced	
21	*drosomycin_g2*	Drosomycin	201	66	PF00304	Gamma-thionin	PYGN01000062	C0J52_03170
22	*drosomycin_g3*	Drosomycin	201	66	PF00304	Gamma-thionin	PYGN01000062	C0J52_03171
23	*drosomycin_g4*	Drosomycin	201	66	PF00304	Gamma-thionin	Unplaced	
24	*drosomycin_g5*	Drosomycin	201	66	PF00304	Gamma-thionin	PYGN01001559	C0J52_12810
25	*drosomycin_g6*	Drosomycin	216	71	PF00304	Gamma-thionin	PYGN01001559	
26	*drosomycin_g7*	Drosomycin	201	66	PF00304	Gamma-thionin	PYGN01001559	C0J52_12811
27	*drosomycin_g8*	Drosomycin	201	66	PF00304	Gamma-thionin	PYGN01001559	C0J52_12812
28	*drosomycin_g9*	Drosomycin	201	66	PF00304	Gamma-thionin	PYGN01001559	C0J52_12813
29	*drosomycin_g10*	Drosomycin	201	66	PF00304	Gamma-thionin	PYGN01002215	C0J52_23105
30	*drosomycin_g11*	Drosomycin	201	66	PF00304	Gamma-thionin	PYGN01002215	C0J52_23106
31	*drosomycin_g12*	Drosomycin	201	66	PF00304	Gamma-thionin	PYGN01002215	C0J52_23107
32	*drosomycin_g13*	Drosomycin	201	66	PF00304	Gamma-thionin	PYGN01002215	C0J52_23108
33	*attacin-like_g1*	Attacin-like	357	118	PF03769	Attacin_C	PYGN01001824	C0J52_26498
34	*attacin-like_g2*	Attacin-like	360	119	PF03769	Attacin_C	PYGN01001824	C0J52_26498
35	*attacin-like_g3*	Attacin-like	357	118	PF03769	Attacin_C	Unplaced	
36	*blattellicin_g1*	Blattellicin	762	253	PF03769	Attacin_C	PYGN01001824	C0J52_26498
37	*blattellicin_g2*	Blattellicin	729	242	PF03769	Attacin_C	PYGN01001824	C0J52_26498
38	*blattellicin_g3*	Blattellicin	804	267	PF03769	Attacin_C	Unplaced	
39	*blattellicin_g4*	Blattellicin	738	245	PF03769	Attacin_C	PYGN01001824	C0J52_26498

Annotation derived from genome sequence (GCA_003018175.1) and transcriptomic analysis of SRR6784710 (Whole body, adult female sample). Some Attacin_C domains bit scores fell below the database's curated model. (L, Length).

In order to revise the annotated AMP coding genes, several *B. germanica* RNA-Seq SRA experiments (PRJNA389591) were screened for their expression using BLASTN and several AMP CDS as queries. Among the SRA runs with abundance of AMP reads, the RNA-Seq run SRR6784710 (whole body, adult female) was selected. Run SRR6784710 was assembled with de novo Trinity^25^ and a transcript database was created.

The annotated genome was compared with the transcript database with the aim of identifying the complete sets of AMP genes for each class. After careful revision, we identified 39 AMP genes (belonging to five types: defensins, termicins, drosomycins, attacins-like and blattellicins), which will be described below. Thirty-four of them were distributed in ten genome scaffolds and five genes were unplaced (Table 1; Supplementary Table 2).

### Defensin AMP genes

Ten annotated AMP CDS with a Defensin domain were used as queries against the SRR6784710 transcript database with BLASTN (e-value = 1.0E−20). All of them produced hits with at least one transcript. In total, 16 different transcripts were identified. The transcript abundance ranged from TPM (transcripts per million transcripts) values of 323.64–0.00.

Information on genome annotation and the assembled transcripts was compared (see Materials & Methods) identifying 16 defensin genes (Supplementary Tables 2 and 3). They received the names of *defensin_g1* to *defensin_g16*, with *defensin_g1* and *defensin_g16* including two alternative isoforms that do not affect the coding region. *Defensin_g1* isoforms i1 and i2 differed in the removal or not of a 3′-UTR intron, whereas the two isoforms of *defensin_g16* differed in the use of different poly(A) signals.

Defensin genes (except *defensin_g1* that was unplaced) were clustered in four scaffolds. The unplaced *defensin_g1* was included because the program identified three transcripts belonging to the cluster TRINITY_DN1123_c0. One of them (corresponding to *defensin_g2*) could be related with gene C0J52_24001 (encoding a hypothetical protein), although we recovered the correct reading frame after the correct placement of the start of the second exon. The other two transcripts showed 100% identity but differed in the alternative splicing of a 453-nt 3′-UTR intron. We considered them isoforms of *defensin_g1*, a different gene of *defensin_g2*, because they differed in seven nucleotides (two in the CDS) plus three indels of different sizes in the 3′-UTR. However, such a sequence was not detected in any scaffold sequence.

The highly expressed transcript (TRINITY_DN13842_c0_g1_i1) was apparently derived from the incorrect assembly by TRINITY of the reads of four different loci in the genome with almost identical sequences (*defensin_g3* to *g6*). Three of them were previously annotated with the locus_tag qualifiers C0J52_27569, C0J52_22338 and C0J52_24004. However C0J52_27569 (gene = DEFI_4 in scaffold PYGN01003429) was a tandem of two genes (*defensin_g3* and *defensin_g4*). An assembly gap overlapping with *defensin_g3* is probably the reason explaining why a single mRNA expanding both genes was annotated in the genome.

Genes *defensin_g7* and *defensin_g8* displayed identical CDS sequences but with several differences in the UTR segments of the mRNA sequences. They were placed in scaffolds PYGN01002380 and PYGN01001185, respectively. Only one of them, *defensin_g8*, was previously annotated as gene C0J52_22336.

*Defensin_g9* corresponds to gene C0J52_24005 encoding Phormicin, a 91-amino-acid protein. The transcript analysis revealed that the encoded protein is shorter (71 amino acids) with a signal peptide sequence of 20 amino acids at its amino-terminus (see below). *Defensin_g10* was also a Phormicin located in a different scaffold, but only the second exon was present in the genome, with the first exon most likely placed in a contiguous 1-kb assembly gap.

*Defensin_g11, g12 and g13* are equivalent to previously annotated genes (Supplementary Tables 2 and 3). *Defensin_g14* is present in scaffold PYGN01001185, but most of the sequence of the second exon is absent because of an assembly gap. The CDS sequences of *defensin_g15* and C0J52_20459 were identical but transcript analysis of *defensin_g15* suggested a two-exon mRNA instead of the three-exon C0J52_20459.

**Figure 1:**
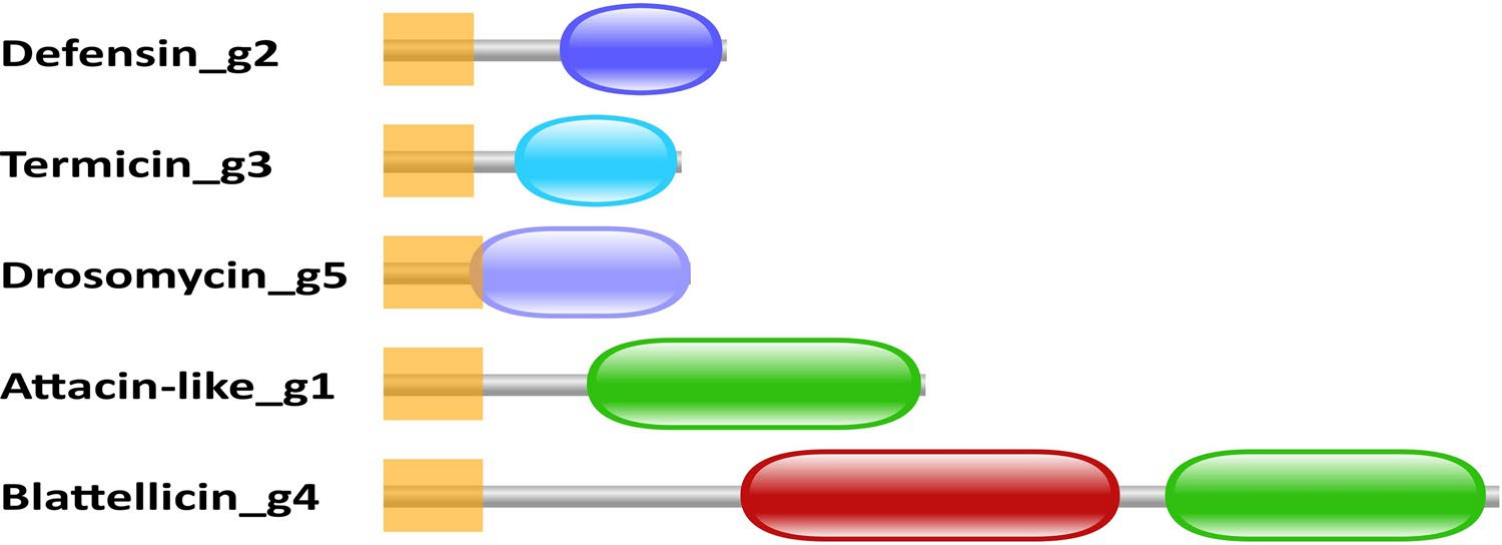
Domain organization in the five types of AMPs of *B. germanica*. One protein of each class is shown. Orange squares are signal peptides. Red oval corresponds to a glutamine/glutamic acid-rich region. Green ovals are Pfam-A domains PF03769 (Attacin_C). Blue ovals (from top to bottom) are Pfam-A domains PF01097 (Defensin_2), PF11415 (Toxin_37) and PF00304 (Gamma-thionin), respectively.

**Figure 2:**
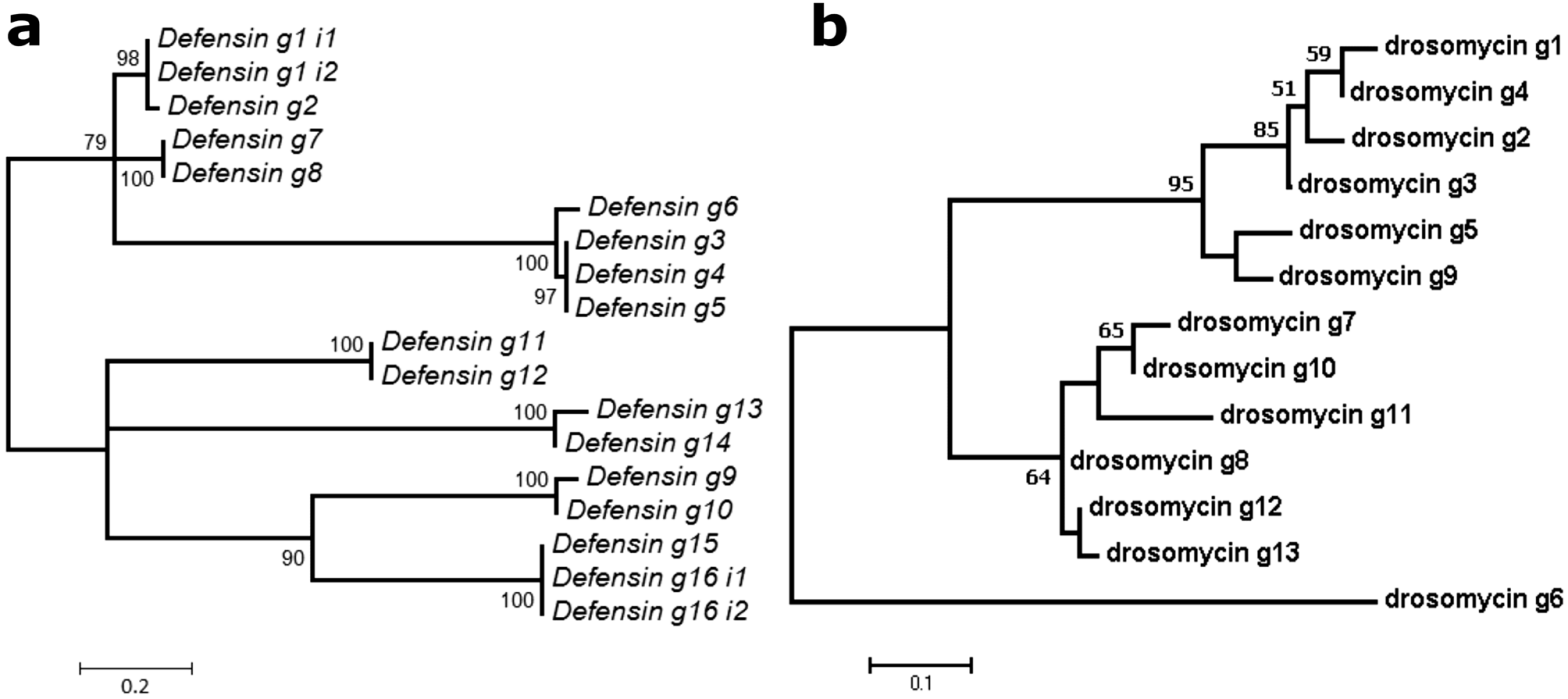
*B. germanica* Defensin and Drosomycin protein phylogenies. (**a**) Maximum likelihood phylogeny of 18 Defensin proteins (derived from transcripts of 16 genes). Model WAG + I with complete deletion. Alignment length 57 sites. Bootstrap replicates 100. Mid-point rooting. (**b**) Maximum likelihood phylogeny of Drosomycin proteins. Model Dayhoff + G with complete deletion. Alignment length 66 sites. Bootstrap replicates 100. Mid-point rooting. Bootstrap values smaller than 50 are hidden.

All Defensins showed signal peptides of 18 to 22 amino acids at the N-terminus and the PF01097 (Defensin_2) domain at the C-terminus (see examples of domain organizations at Fig. 1). The length of the amino acid chain ranged from 63 to 81 residues with an average of 72 amino acids. Although some Defensin proteins were identical, the average number of pairwise differences was high (29 amino acids). An inferred maximum likelihood phylogeny showed their distribution in seven clusters (Fig. 2a). A logo of the protein alignment of the Defensin proteins shows the hydrophobic N-terminal sequence as well as the Defensin_2 domain (C-terminus) with the six conserved cysteine residues (Supplementary Fig. 1).

A comparison among the levels of transcription of the 16 defensin genes was estimated using a BLASTN strategy based on BLASTN searches with nucleotides 41–190 of each CDS. All 150-nt sequences were different in, at least, one nucleotide, except *defensin_g3* and *g5* that were identical and the transcription level could not be assigned to a specific gene (Supplementary Table 3). Based on the TPM values estimated by TRINITY and the transcription levels estimated by this BLAST strategy, we observed that in this female adult run, *defensin_g15* and *g16* (encoding Defensin-like proteins), *g9* and *g10* (encoding Phormicin) and *g1*, *g2*, *g3* and *g5* (encoding Tenecin-1 proteins) are the most highly expressed defensin genes (Supplementary Table 3).

Using a TBLASTN strategy, defensins transcripts were searched in 45 species covering the order Blattodea^26^ (Supplementary Table 4). Forty-four species contain defensin transcripts (range 1 to 9).

### Termicin AMP genes

Three genes encoding small proteins with the Pfam domain PF11415 are annotated in the genome (Supplementary Table 1). BLASTN searches against the SRR6784710 transcript database gave hits with only two very similar transcripts. The first transcript, TRINITY_DN10017_c0_g1_i1, displayed one single difference with either C0J52_00758 or C0J52_26761 at the CDS sequence but several at the remaining mRNA sequence, suggesting two independent genes in the genome. The second transcript, TRINITY_DN10017_c0_g2_i1, was 100% identical with both CDS and mRNA from C0J52_26762, indicating a third termicin gene. The three encoded proteins are almost identical with a single S/A difference at site 13 (Supplementary Fig. 1). A signal hydrophobic peptide is predicted between amino acids 1 and 19 and the Toxin_37 domain (PF11415) between amino acids 30 and 63 (Fig. 1). Based on the TPM values estimated by TRINITY and the transcription levels estimated by BLASTN (a 150-bp segment covering four polymorphic sites in the termicin CDS), we may conclude that *termicin_g3* (C0J52_26762) is the most highly expressed termicin gene (Supplementary Table 5).

Termicin mRNAs were detected in 29 Blattodea species belonging to the different taxonomic families (Supplementary Table 4). Their absence was frequent in species from Corydioidea, suggesting the potential loss of this type of gene, although the lack of expression in these samples may not be ruled out.

### Drosomycin AMP genes

Ten genes encoding proteins with the domain Gamma-thionin (PF00304) are annotated in three scaffolds of the *B. germanica* genome. These antifungal proteins receive the name of Drosomycins. BLASTN searches of the annotated CDS against the SRR6784710 transcript database identified only six transcripts including the complete CDS and two insignificant transcripts covering only a CDS segment.

The comparison of the annotated CDS and those derived from these transcripts revealed that only three annotated genes (C0J52_03170, C0J52_03171 and C0J52_12810) were equivalent to three of these transcripts (the former with 2 nucleotide differences). They were annotated as *drosomycin_g2*, *g3* and *g5* (Supplementary Tables 2 and 6). One of the three remaining transcripts, corresponding to *drosomycin_g*6, could be placed in the genome, with a few nucleotide differences, in a non-annotated segment. Finally, the sequences of the other two transcripts were not detected in the genome, although their CDS sequences were highly similar to C0J52_03170 (with 6 and 8 nucleotide differences). These differences suggest that they are not alleles but independent genes and we annotated them as *drosomycin_g1* and *g4* (Supplementary Tables 2 and 6).

On the other hand, six annotated genes with locus_tags, C0J52_12811-13 and C0J52_23105-08 were not detected in the adult female transcriptome, but they seem to be expressed in other developmental stages. They were annotated as *drosomycin_g7* to *g13*.

A phylogeny of the 13 Drosomycin proteins showed that *defensin_g6* was the most distant gene, while the other 12 genes formed two clusters of six genes each. Genes *drosomycin*_*g1* to *g5*, expressed in adult females, plus the non-expressed *drosomycin_g9* formed one well-supported clade while the other six non-expressed genes formed the other (Fig. 2b).

An estimation of the transcription level revealed that *drosomycin_g5* (C0J52_12810) was the gene with the highest expression, with 86.1% of the drosomycin reads for this segment derived from it (Supplementary Table 6).

Twelve out of 13 encoded proteins were 66 amino acids long. Drosomycin_g6 was 71 amino acids long due to the presence in the middle of the protein of additional amino acids derived from two indels (sites 25–26 and 36–38 of the alignment). Among the observed residues, the most remarkable feature in the encoded proteins is the presence of eight conserved cysteines^27^ (Supplementary Fig. 1). All Drosomycins display a signal hydrophobic peptide at the N-terminus and the PF00304 domain (Gamma-thionin) at the C-terminus (Fig. 1).

Drosomycin mRNAs were detected in 24 Blattodea species but they were absent in species of Isoptera and their close relative *Cryptocercus wrighti* (Supplementary Table 4). The same fact was detected in the clade Corydioidea, suggesting that termites and other Blattodea may have lost this type of AMP gene.

### Attacin AMP genes: attacin-like and blattellicins

Up to four regions with some similarity with the Attacin_C domain (PF03769) were detected in the 47-kb-region spanning the C0J52_26498 gene placed in contig PYGN01001824. After a preliminary analysis of the assembled transcriptome, more than ten mRNA sequences were identified. They resemble complete or partial sequences of mRNAs belonging to two types of attacin genes. The first type includes genes encoding typical Attacin proteins (around 120 amino acids) with a signal peptide at the N-terminus and the Attacin_C domain at the C-terminus, which were named as attacin-like genes. The second type was very different, because it included a long stretch of glutamine/glutamic acid residues. Since they seemed an apparent evolutionary innovation in *B. germanica*, we called them blattellicins.

Three attacin-like transcripts were detected in the transcriptome (Supplementary Tables 2 and 7). They contained coding sequences of 357–360 nucleotides (118–119 encoded amino acids). They received the names of *attacin-like_g1* to *attacin-like_g3*. The extraction and assembly of the reads for these mRNAs confirmed their existence, but suggested the possibility of a fourth gene. *Attacin-like_g3A* and *attacin-like_g3B* display only two differences, the deletion of a 9-nucleotide-segment in the 5′UTR of *attacin_g3B* and a synonymous difference at CDS position 288 (the inclusion of the sites for the two differences in a read was very infrequent considering that the length of a read is 301 nucleotides). Because there were only two differences and they were unplaced in the genome, we considered that they were alleles of the same gene.

*Attacin-like_g1* CDS was relatively similar to *attacin-like_g3* CDS with 9–10 differences. However, they were sufficiently different to be considered independent loci. *Attacin-like_g2* was the most divergent gene with 85–88 differences and an extra codon against the others. Only the sequences of *attacin-like_g1* and *g2* were located in the genome (Supplementary Tables 2 and 7).

The annotation of blattellicins was much more complicated. After a preliminary analysis, a long CDS (> 250 codons) with a curious structure was observed. It started with the hydrophobic signal peptide at the N-terminus, followed by a long Glx-rich segment in the middle (> 70 residues, mainly glutamines and glutamic acids) and a C-terminal Attacin domain (Fig. 1).

Up to 13 mRNA transcripts (all of them containing incomplete CDS segments) involving this type of sequences were detected. The main reasons were that the presence of several blattellicin genes and the long Glx-rich regions drastically affected the assembly of the transcriptome. This fact probably took place during the assembly and annotation of *B. germanica* genome^5,6^.

The 5′ sequence of a blattellicin CDS was used as query to identify with BLASTN those reads derived from the expression of blattellicin genes in the run SRR6784710. After extraction and assembly, four different starts of blattellicin genes were revealed, with a range of 7 to 18 pairwise nucleotide differences in the 5′ of mRNAs. These four mRNA starts were used to recruit the remaining gene sequences until CDS completion.

Most of the CDS sequence for *blattellicin_g1* could be identified in the genome, although around 200-bp were absent due to two assembly gaps (Supplementary Tables 2 and 7). For the others, only the first coding exon of *blattellicin_g2* and *g4* could be unequivocally assigned to a specific contig segment, although hits for other segments of the CDS were also detected but without 100% identity. No identical sequence to *blattellicin_g3* first exon could be identified in the genome. The most feasible explanation is that the four blattellicin genes are present in tandem copies in the genome but their special central repeat structure prevents correct assemblies in either genome or transcriptomes, except if manual inspection of the alignments is performed. Furthermore, variations in Glx codon copy number in the population cannot be ruled out.

We detected that blattellicins were expressed at a higher level than attacin-like genes, with *blattellicin_g4* being the most highly expressed in this transcriptome (Supplementary Table 7).

**Figure 3:**
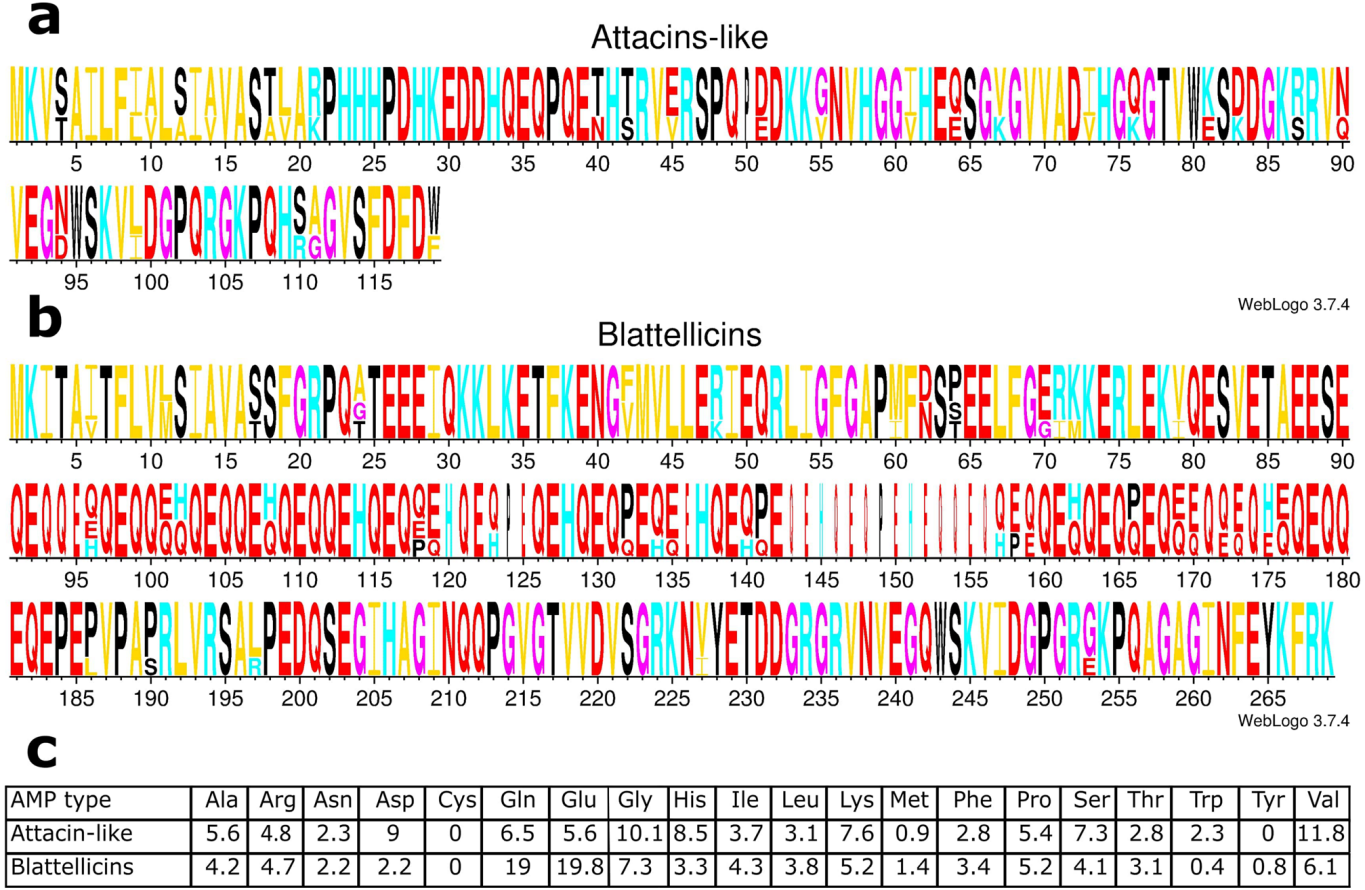
Logos of alignments and amino acid composition of *B. germanica* Attacin-like and Blattellicin proteins. (**a**) Logo of the alignment of three Attacin-like proteins. (**b**) Logo of the alignment of four Blattellicins. (**c**) Average amino acid composition (%) of Attacin-like and Blattellicin proteins.

Logos of the protein alignments for the three Attacin-like and the four Blattellicin proteins of *B. germanica* revealed a small segment of negatively charged amino acids in Attacin-like proteins and a long segment in Blattellicins (Fig. 3).

Attacin mRNAs were detected in most Blattodea species (Supplementary Table 4). Hits for blattellicins did not cover the Glx region but only the attacin_C domain. To understand the evolutionary history of attacin-like and blattellicin genes in *B. germanica*, we extracted the attacin transcripts from seven Blattellinae TSA projects^26^ (*Symploce* sp*. AD-2014, Loboptera decipiens, Episymploce sundaica, Ischnoptera deropeltiformis, Paratemnopteryx couloniana, Lobopterella dimidiatipes, Asiablatta kyotensis*). These transcriptomes come from adult whole bodies except *I. deropeltiformis* (without information about developmental stage). They may potentially cover all attacins genes for each genome, although the possibility of genes without expression cannot be discarded. The largest number of attacin genes was three in *E. sundaica*. Two genes were observed in *L. decipiens*, *Symploce sp.* AD-2014 and *A. kyotensis*, although in the former one of the copies was incomplete and very divergent, probably a pseudogene, while in the latter, the two copies were a few codons incomplete at 5′-end of the CDS. The SRA project was screened for reads covering the start of the CDS and, based on those recovered, one was completed and, in the other, only four codons were missed. The remnant species contained a single gene copy. In addition, in order to use as an outgroup, the only-one-detected in *P. americana* was extracted.

**Figure 4:**
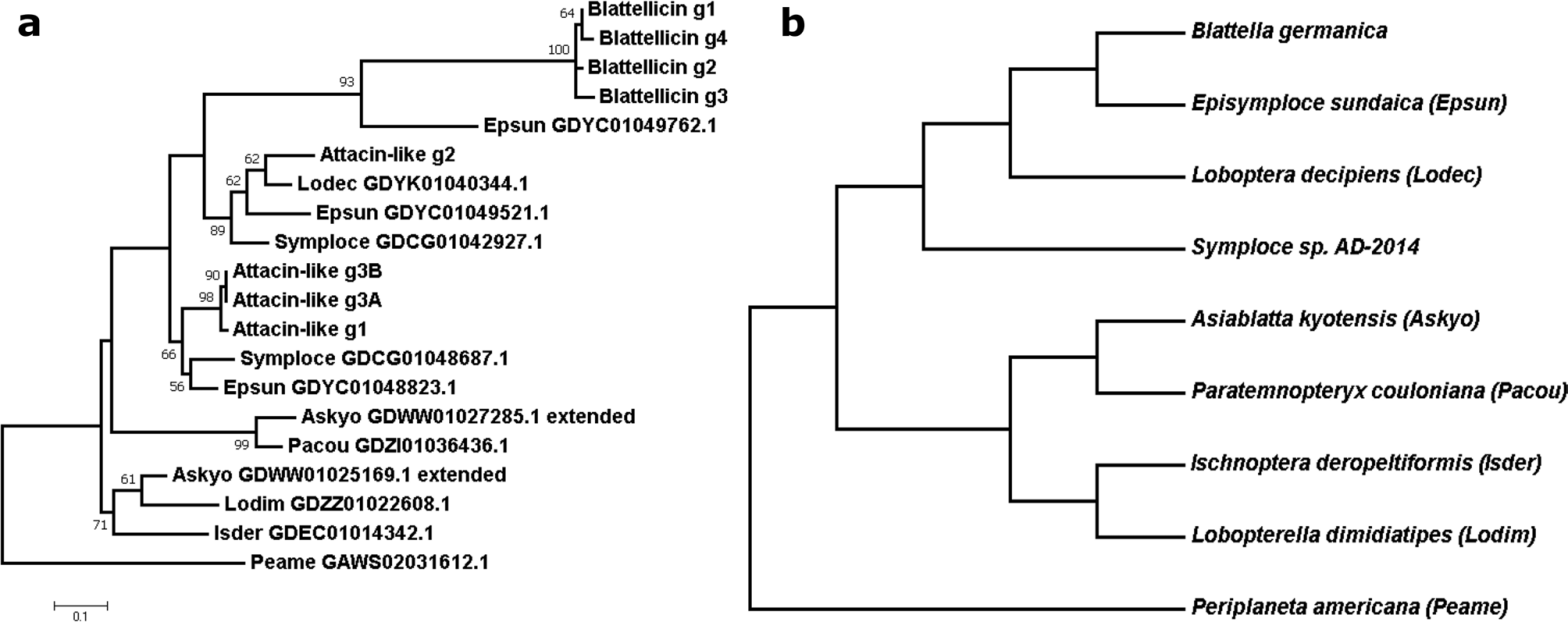
Attacin-like and Blattellicin protein phylogeny in Blattellinae. (**a**) Maximum Likelihood phylogeny of proteins containing Attacin_C domain in the subfamily Blattellinae. Model LG + G with complete deletion. Alignment was trimmed to join the N-terminal signal peptide plus the C-terminal attacin_C domain (length 103 sites). *P. americana* was used as an outgroup. Bootstrap replicates 100. Bootstrap values smaller than 50 are hidden. All species names are abbreviated (see codes in the right topology), except *Symploce* sp. Those without abbreviations are proteins from *B. germanica*. (**b**) Taxonomic relationships according to^26^.

A phylogeny was performed with a trimmed alignment (103 sites) (Fig. 4). The short length of the sequence alignment prevented high bootstrap values in most nodes and hampered determining with complete confidence the evolutionary history of this gene family. However, several facts are observed from the phylogeny. First, attacin-like genes are the ancestral gene type. Some Blattellinae species contain only one or two genes. In the case of the clade of *B. germanica*, *E. sundaica*, *L. decipiens* and *Symploce* sp*. AD-2014,* the duplication of an ancestral attacin-like gene took place before their divergence, resulting in the appearance of *attacin-like_g1* and *g2* types. Although *L. decipiens attacin-like_g1* was not included in the phylogeny, an incomplete and divergent copy of a transcript of this type (GDYK01026461.1) is detected, probably derived from a pseudogenized copy.

The origin of blattellicins seems to be very recent. Although not supported by a significant bootstrap value, potentially an ancestral *attacin-like_g2* type gene was duplicated and one of the copies, after a fast evolution, generated blattellicins. The duplication took place prior to the divergence of *E. sundaica* and *B. germanica*. The protein in the former is apparently a pre-Blattellicin, including some of the new characteristics of Blattellicins, such as large size (182 residues) and a few extra amino acids at the C-terminus (RK in *B. germanica* and GKGK in *E. sundaica*). However, the main characteristic of Blattellicins, the long poly-Glx region, is absent, although *E. sundaica* pre-Blattellicin includes a seven-glutamic-acid track (with an A in the middle) close to the start of the attacin domain.

### AMP expression in *B. germanica*

To determine the expression of AMP genes in *B. germanica* tissues, developmental stages or sexes, we selected the CDS of 17 AMP gene types *(defensin_g2*, *g3*, *g7*, *g9*, *g11*, *g13* and *g15*; *termicin_g1*; *drosomycin_g1*, *g5*, *g6, g11* and *g12*; *attacin-like*_*g1* and *g2*; *blattellicin_g1* and *g4*). They are sufficiently different to avoid important cross results among those selected of the same group. However, due to the high similarity of the CDS of some genes from the same family, the obtained values showed the expression of the sets of genes with almost identical sequences (for example, the three termicin genes or *attacin-like_g1* and *g3*).

**Figure 5:**
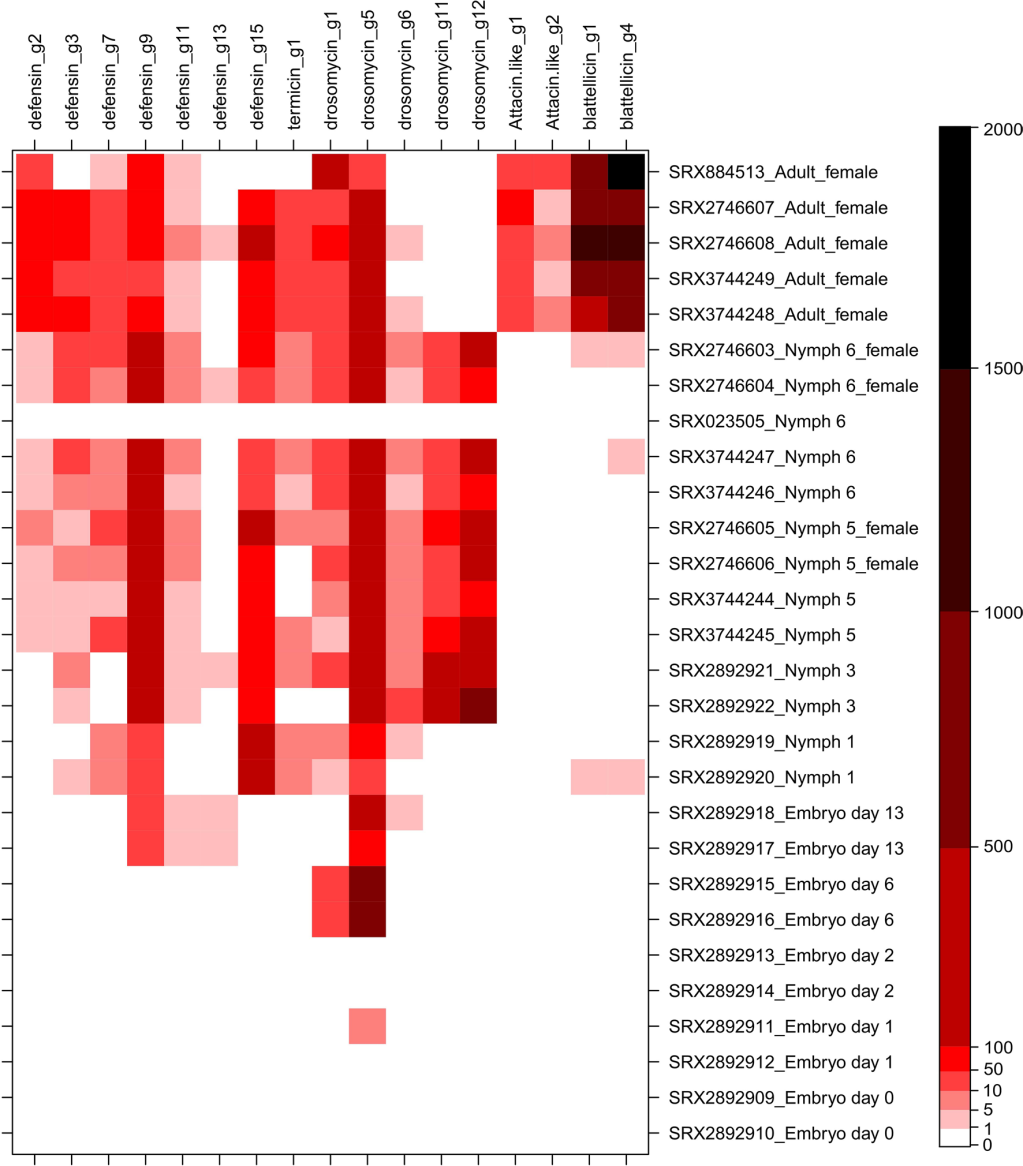
Gene expression of 17 AMP genes in whole bodies of *B. germanica*. Heatmap analysis illustrating the abundance of transcripts for 17 selected AMP genes in 28 Sequence Read experiments corresponding to whole bodies from diverse developmental stages of *B. germanica* with indication, in some cases, of the sample sex. Values were estimated as the quotient between the number of reads producing a hit with an e-value smaller than 1.0E−40 (using the complete CDS sequences as queries in BLASTN searches) and the size in Gb of the SR experiment.

Expression levels were estimated with a BLASTN strategy as number of hits/Gb of SR experiment (Supplementary Table 8). The heatmap analysis of 28 whole body SR experiments corresponding to samples from different developmental stages (Fig. 5) revealed several conclusions. First, adult females displayed high expression of most AMP genes, although the most relevant was the highest expression of *blattellicin_g1* and *g4*. Some drosomycins were also highly expressed, specially, *drosomycin_g5*. The expression of some genes was connected with development (see, for example, the absence of expression of *drosomycin g11* and *g12* in adult females but high expression in nymphs). Among defensins, the most highly expressed during most developmental stages were *defensin_g9* and *g15*. The expression of *defensin g2* and *g3* was higher in adult females than in nymphs. *Termicin_g1* displayed low expression in nymphs and adults. Attacin-like genes were also expressed in adult females, with *attacin-like_g1* values higher than those of *attacin-like_g2*, which was in agreement with previously described results (Supplementary Table 7), also considering that the detected hits for *attacin-like_g1* probably comes from *g1* and *g3* genes.

In general, AMP genes display an increase in expression as development progresses to adult forms. Unfortunately, no SR experiment for exclusively adult males was deposited in SRA database, although some mixed males and females samples are reported (Supplementary Table 8).

We also analysed the expression of these 17 AMP genes in some transcriptomic SR experiments in which samples come from one single tissue, a part of the body or a mixture of several tissues (Supplementary Table 8). In general, *drosomycin_g5 and defensin_g9* seem to be expressed in most of these samples. In two experiments from male adult heads, several AMP genes were expressed to a relevant level, including *defensin_g7* and *g9*, *drosomycin_g5* and *attacin-like_g2*. In general, the level of expression in these samples is much smaller than those coming from whole bodies. This leads us to propose that other parts of the body different from fat body, ovaries or epidermis are responsible for the high expression levels observed in whole body adult females (Fig. 5).

No expression of *blattellicin_g1* and *blattellicin_g4* was observed in any tissue or part of the body sample, except an almost undetectable expression in one non-fecundated eggs sample, probably due to contamination with female tissues.

## Discussion

Estimating the number of AMP genes in any species is a complex task due to their small sizes, to the evolutionary dynamics of these gene families with frequent gains and losses and with redundancy and neofunctionalization^12,14^ and to the difficulties of the treatment of repeats in the genome assemblies^28^. In this work, we have detected in *B. germanica* an arsenal of genes encoding AMPs larger than initially annotated, including some no yet described (Table 1 and CDS and protein sequences in Supplementary Information). However, we cannot discard the presence of immune taxonomically restricted genes encoding divergent or non-characterized antimicrobial domains, as observed in other species^29,30^.

When the set of 11 AMP genes of the American cockroach *P. americana*^4^ was compared with the AMP gene set annotated in *B. germanica*, the expansion of some key protein families, such as the anti-fungal drosomycins were described^5^. A deeper analysis (present study) has revealed the existence of 13 drosomycin and 16 defensin genes. Some are very similar, almost identical, but others display differences in more than 50% of the encoded amino acids (Fig. 2). Two additional families, termicins and attacins, were detected. Three, almost identical, termicin genes are detected in *B. germanica* in front of two in *P. americana*. Because of their high identity, two possibilities exist: they are recent duplications, or they evolved by gene conversion. We have divided the gene family of attacins in attacin-like (three genes) and blattellicins (four genes). Although initially two Attacin domains were annotated in Diptera (the Attacin_N and the Attacin_C, at each end of the proteins), Attacin protein alignments of distant insects suggested that Attacin_N is an artefact^12^ and that Attacin_C is the only domain common to Attacins. Both *B. germanica* Attacin-like proteins and Blattellicins contain this domain, although these proteins differ drastically in size and in the presence of the long Glx-rich region (Fig. 1). Two additional gene types, one AFP (antifungal peptide) and a Pro-rich peptide, detected in *P .americana*^4^, seem not to have a counterpart in *B. germanica*.

The search of the five types of *B. germanica* AMP genes in TSA projects from Blattodea (Supplementary Table 4) revealed that four of them (defensins, termicins, drosomycins and attacins) were widely distributed against the different taxonomic suborders, superfamilies or families. However, it was relevant the loss of the antifungal drosomycin genes in the termites and the wood roach *Cryptocercus*, which would be related with the different types of interactions with fungal symbionts acquired during evolution, as the domestication of *Termitomyces* fungi in Macrotermitinae^31^. However, this loss may be compensated by the presence of several termicin genes (Supplementary Table 4), potentially displaying strong antifungal activity^32^. Expansions of the termicin gene family were observed in two termite species affected by fungal contamination^33^.

By using BLASTP searches against UniProtKB database limited to Insecta (data not shown), we detected Defensin proteins mainly in Blattodea, Coleoptera, Diptera, Hemiptera and Hymenoptera and a few cases in other taxonomic orders. Termicins were restricted to Blattodea. Drosomycins were detected in Blattodea, Coleoptera and Diptera. Similar sequences to Attacins were detected in Blattodea, Diptera, Hemiptera, Orthoptera and Phasmatodea.

It has been suggested that defensin genes were already present in the last common ancestor of all insects^12^. Attacins were also considered ancient genes and their origin was placed before de divergence of Palaeoptera and the clade Polyneoptera/Holometabola^12^. The origin of drosomycins is controversial because they have been found in plants and in Ecdysozoa (Arthropoda, Nematoda and Tardigrada) and it was suggested that the ancestor of ecdysozoans acquired this type of gene from plants through a single horizontal gene transfer event^34^. Finally, termicin genes are apparently an innovation that predated Blattodea divergence.

The microbial targets for each type of *B. germanica* AMP are not characterized but, based on a CRISPR gene editing study in *Drosophila*, we know that some AMPs have broad taxonomic ranges and others very narrow^10^. While two *D. melanogaster* Drosomycins inhibit the growth of several fungal species, they are inactive against all the bacterial species tested^35^. Termicins display strong antifungal activity in termites and very low anti Gram-positive bacteria^32^. Activity assays of Defensins (PF01097) in species belonging to different insect orders (Diptera, Coleoptera, Hymenoptera, etc.) revealed effects mainly on Gram-positive species and in a lesser extent on Gram-negative and fungal species (see Function section in UniProtKB database files). In *Drosophila*, it was shown than Attacins are mainly affecting anti Gram-negative bacteria^36^ and they are induced after immune challenge with a Gram-negative pathogenic *Serratia*^37^.

An estimation of the isoelectric points (pI) of complete *B. germanica* AMP proteins reveals that only Defensins and Drosomycins are cationic. Blattellicins and Termicins are anionic while the pI of Attacin-like proteins is around 7. The protein Defensin 2 from the tick *Amblyomma hebraeum* with a pI 4.44 showed activity against *Escherichia coli* and *Staphylococcus aureus*^38^. Non-cationic Defensins were also detected in some lepidopteran species such as *Spodoptera littoralis*^39^, *Bombyx mori*^40^ and *Galleria mellonella*^41^. In the latter species, anionic peptide 2 (a mature peptide of 60 residues) contains 13.3% and 8.3% of glutamic acid and glutamines, respectively. A recent study showed that this peptide decreases the survival rate of *Candida albicans* through its action on the cell wall, and bioinformatics analysis suggest that this action is mediated by the presence of amphipathic alpha-helices with exposed positively charged lysine residues, regardless of its anionic character^7^. Termicin structure from the termite *Pseudacanthotermes spiniger* displays a hydrophobic face formed by a large aggregate of hydrophobic residues and a hydrophilic face, including three charged residues^42^. Because anionic *B. germanica* AMPs contain positively charged amino acids in relevant proportions (Termicins *ca*. 6%, Blattellicins *ca*. 13% and Attacin-like *ca*. 20%), we cannot discard that they contain protein 3D regions with abundance of positively charged residues, or they are able to produce cationic mature peptides after proteolytic cleavage.

The most relevant fact of Blattellicins, the new type of Attacins, is the long Glx-rich region, whose function is unknown but polyglutamine (polyQ) tracts has been detected in some transcription factors such as TATA-binding proteins^43^, and several human proteins, such as Ataxins or Huntingtin, are involved in diseases related with glutamine expansions. PolyQ containing proteins tend to produce fibrillar aggregates and to bind lipid membranes in a polyQ-length dependent manner^44^. Prediction of the secondary structure of Blattellicins renders several contiguous alpha-helices covering most of the segment between the signal peptide and the Attacin_C domain (data not shown). The presence of charged amino acids (Glu) may aid in solubility as previously described for polyQ peptides^44^. An analysis of the amino acid composition of 398 proteins with an Attacin_C domain in insects (UniProtKB database) did not show high percentages (> 10%) of either glutamine or glutamic acid in most of them. High percentages were detected in only 4 proteins and the only relevant sequence was a 578-residues Attacin C domain-containing protein of unknown function from *Anopheles stephensi* containing long stretches of glutamine residues and some glutamic acids in the proximity of the attacin_C domain (A0A182Y178). AMP gene expression during development is not homogeneous in *B. germanica*, with genes expressed during the whole development, genes mainly restricted to adults and genes expressed only during nymphal stages (Fig. 5). Previously, the enriched expression in *B. germanica* adults of genes involved in immune defence such as those containing the Defensin_2 domain was reported^45^. The level of expression of *dorsal* during development of *Tenebrio molitor*, which is involved in the Toll immune pathway, is also variable with the highest levels in young adults^46^. In *D. melanogaster*, the expression analysis of seven drosomycin genes in whole bodies along development, showed some genes expressed from larva to adult and others only expressed in some developmental stages^35^.

Because the number and types of *B. germanica* analysing tissue-specific transcriptomic projects is reduced (Supplementary Table 8), we do not know which are the main tissues in both nymphal and adult stages where each AMP gene is expressed. A broader study should be performed to discriminate among some potential tissues where components of the insect humoral immune system are known to be expressed^46–49^, such as fat body, haemocytes, gut, salivary glands, trachea, Malpighian tubules, and integument. Fat bodies have been reported to display high expression level for some AMP types such as defensins^49,50^ and attacin^51^. Defensin genes from adult *A*. *stephensi* are expressed in fat body, midgut, haemolymph and salivary glands with the high level of expression at fat body^49^. On the contrary, a defensin gene from *Bemisia tabaci* was more highly expressed in adult midgut or salivary glands than in fat bodies^52^ and a type of attacin gene was highly expressed in adult Malpighian tubules of the stick insect *Carausius morosus*^53^. It was also reported constitutive expression of a termicin gene in adult haemolymph and salivary glands from the termite *P. spiniger*^32^.

Of special interest is the expression in hindgut, along the development, because the rich and variable microbiota that is acquired after birth may be, somehow, controlled by the host. Moreover, for a correct relationship with *Blattabacterium*, its ancient endosymbiont, *B. germanica* could be using one of its AMP genes, expressing it differentially in the fat body bacteriocytes and in the rest of the body, as it is the case in *Sitophilus zeamays* with *coleoptericin A*^14,54^. Because Coleoptericin and Attacin contain a related domain, we can imagine that one of the Attacin-like proteins or the Blattellicins may be in charge of this function. In the louse fly *Melophagus ovinus*, attacin is one of the genes upregulated in midgut but downregulated in bacteriocytes^55^, probably related to the relationship with its obligate endosymbiont *Arsenophonus melophagi*.

Finally, knowing the tissues where Attacin-like proteins and Blattellicins are expressed will require additional RNA-Seq studies including both developmental stages and specific tissues. The expression of two attacin-like genes in adult male heads may be related with the initial parts of the gut, while the absence of Blattellicin synthesis in fat body, ovaries and epidermis (Supplementary Table 8) suggests their synthesis in other tissues or cells, such as the different parts of the gut, Malpighian tubules, salivary glands or haemocytes.

## Methods

### Sequence data and strategy for AMP gene set revision

The genome sequence of *B. germanica* (Orlando Normal from American Cyanamid) was obtained from GenBank assembly accession GCA_003018175.1 (WGS Project PYGN01)^6^. The sequence data of run SRR6784710 (SRX3744248), belonging to *B. germanica* whole body adult female 5-days old sample^45^, was used to produce an assembled transcriptome. To integrate the genome annotation and the assembled Trinity transcripts, each transcript was aligned with the genome using BLASTN against *B. germanica* genome project (GCA_003018175.1). Transcripts were also aligned to scaffold sequences with the program Spidey in the platform UGENE^56^, to check for the correct splicing, to compare potential alternative spliced transcripts, and to identify partial genes.

Information from available *B. germanica* transcriptomic projects (Supplementary Table 8) was extracted from NCBI.

A TBLASTN strategy was used to identify transcripts producing hits using *B. germanica* AMPs as queries (e-value = 0.001) in 44 Blattodea SRA projects plus the annotated mRNAs of *Z. nevadensis*^26^ (Supplementary Table 4). Sequences of *attacin* CDS were obtained from the transcriptome shotgun assemblies of seven Blattellinae TSA projects (*Symploce* sp*. AD-2014, L. decipiens, E. sundaica, I. deropeltiformis, P. couloniana, L. dimidiatipes, A. kyotensis*)^26^.

### Transcriptome assembly

The transcriptome of *B. germanica* was assembled from the SRA project SRR6784710. Fastq files obtained using SRA toolkit fastq-dump tool (http://ncbi.github.io/sra-tools/) were filtered with PRINSEQ^57^ to obtain sequences with a mean quality of 20 Phred and a minimum length of 100 bases. Illumina adapters were removed using cutadapt^58^. The assembly was performed with de novo Trinity^25^ using default parameters.

### Transcriptome level estimations

Although, TFM (transcripts per million transcripts) values were estimated for the transcriptome assembled with de novo Trinity (Supplementary Tables 3, 5, 6 and 7), because the integrated revision of genome annotation and transcriptome rendered a slightly different set of AMP genes, we developed an additional strategy. To estimate the level of expression of genes in adult females, BLASTN searches were performed using CDS segments of 150 nucleotides length as queries against SRX3744248, and the number of hits covering 100% of the query with 100% identity were filtered and counted. In drosomycins and attacins/blattellicins, we use the first 150 nucleotides of each CDS but, in the defensins and termicins, we selected a different segment trying to discriminate the maximum number of genes but, in a few cases, it was not possible (Supplementary Tables 3, 5, 6 and 7). For each gene, the expression value was calculated relative to its type of AMP gene family, as a percentage over the total number of hits for the set of genes of its type (Supplementary Tables 3, 5, 6 and 7).

To estimate the level of expression in SR experiments of whole bodies during development or in tissues or body parts (Fig. 5 and Supplementary Table 8), BLASTN searches were performed using the complete CDS of 17 AMP genes as queries against each SR experiment. Gene expression was estimated as the quotient between the number of reads producing a hit with an e-value smaller than 1.0E−40 (maximum of 5000 results) and the size in Gb of each SR experiment. An additional normalization (hits/Gb and length CDS) was calculated, but, although the long blattellicins displayed comparatively lower levels, they were already the highly expressed AMP genes in whole body adult females. Because several AMP genes belonging to the same family are very similar, the reported expression values may include hits belonging to additional genes (for example, the expression value of *termicin_g1* is probably covering the expression of the three termicin genes). In general, the 17 AMP genes were selected based on its position in the AMP phylogenies, trying to select one gene for each phylogenetic group. In general, the selected genes, in each group, were those with the highest expressions in adult females, except *drosomycin_g11* and *g12* without expression (Supplementary Tables 3, 5, 6 and 7). Heatmaps were plotted using R 3.6.0^59^ and package lattice^60^ (v0.20-41).

### Sequence alignment and phylogenetics

BLAST searches were performed at the NCBI server or locally with BLAST+ software^61^. Multiple sequence alignments were performed in MEGA7^62^ and in Unipro UGENE^56^ with the program MUSCLE^63^. For phylogenetic analyses, AMP protein alignments were manually revised according to the domain alignments generated with HMMER (https://www.ebi.ac.uk/Tools/hmmer/search/hmmscan). The evolutionary history was inferred by using the Maximum Likelihood method based on the estimated best protein evolutionary model. For Attacins (including Blattellicins), the phylogeny was inferred using a trimmed alignment corresponding to the concatenation of the first 22 amino acids (20 amino acids of the signal peptide plus two extra amino acids, being the last one proline) plus 81 sites of the C-terminal attacin domain (domain sequence lengths range 74–81).

### Domain organization and signal peptide

The modular structure of AMP proteins (PFAM domains and signal peptides) were identified online (https://www.ebi.ac.uk/Tools/hmmer/search/hmmscan). Domain architecture images were generated in PFAM (https://pfam.xfam.org/generate_graphic). Logos of aligned AMP proteins were generated with WebLogo 3 (http://weblogo.threeplusone.com).

## Supplementary information

Supplementary information.

## Data Availability

*B. germanica* CDS and protein sequences of the described AMPs may be found in Supplementary Information.
