# Supplementary material for: Blattella germanica displays a large arsenal of antimicrobial peptide genes
Source: Sci Rep. 2020 Dec 3;10:21058. doi: 10.1038/s41598-020-77982-3 (PMC7712779; doi:10.1038/s41598-020-77982-3)
Supplement: Supplementary file 1 — Supplementary information. [file 41598_2020_77982_MOESM1_ESM.pdf]

Supplementary Information

Supplementary Figure 1

Supplementary File 1: *B. germanica* CDS sequences

Supplementary File 2: *B. germanica* protein sequences

Supplementary Tables 1 to 8

Blattella germanica displays a large arsenal of antimicrobial peptide genes

Francisco J. Silva[1,2*], Maria Muñoz-Benavent[1], Carlos García-Ferris[1,3] and Amparo Latorre[1,2]

[1]Institute for Integrative Systems Biology (I2SysBio), University of Valencia and CSIC, Paterna, Spain
[2]Genomics and Health Area, Foundation for the Promotion of Sanitary and Biomedical Research, Valencia, Spain
[3]Department of Biochemistry and Molecular Biology, University of Valencia, Valencia, Spain

[*]Corresponding author: francisco.silva@uv.es

Supplementary Figure 1

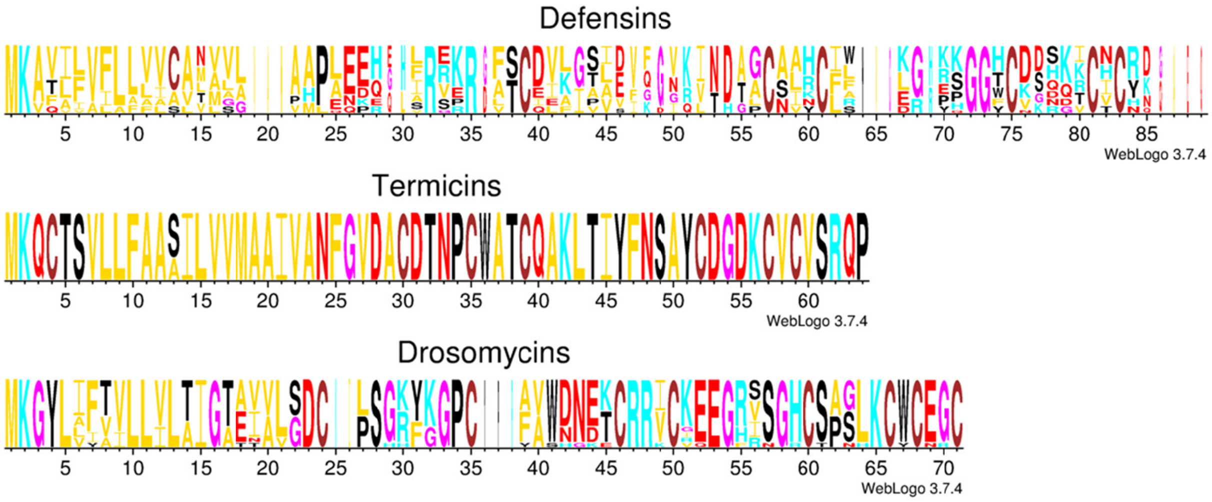

Supplementary Figure 1. Logos of protein alignments of *B. germanica* AMPs containing a Pfam domain of Clan CL0075. From top to bottom, Defensins, Termicins and Drosomycins. Colour codes: AFILMV (gold), C (brown), DEQN (red), KRH (cyan), G (fuchsia), PSTWY (black).

Supplementary File 1: **B. germanica** *CDS sequences*

```
>CDS_defensin_g1_i1
ATGAAGTCCGTTTTGTTGGTATTTCTCCTGGTTGTTTGCGCCGTGGTGTTGGCTGCTCCACTAGAAGAGCATATAAGAAGGAAAAGATTTACGTGTGATGTCCTGGGTTCG
ATTGAGGTTCAAGGTAACCGAATTAATGATGCAGCGTGCGCCTTTCATTGCCTTTGGAAAGGAGAATCTGGAGGATGGTGCGATGACCAAAAGCGGTGCAACTGTCGGGAT
TAA
>CDS_defensin_g1_i2
ATGAAGTCCGTTTTGTTGGTATTTCTCCTGGTTGTTTGCGCCGTGGTGTTGGCTGCTCCACTAGAAGAGCATATAAGAAGGAAAAGATTTACGTGTGATGTCCTGGGTTCG
ATTGAGGTTCAAGGTAACCGAATTAATGATGCAGCGTGCGCCTTTCATTGCCTTTGGAAAGGAGAATCTGGAGGATGGTGCGATGACCAAAAGCGGTGCAACTGTCGGGAT
TAA
>CDS_defensin_g2
ATGAAGGCCGTTTTGTTGGTATTTCTCCTGGTTGTTTGCGCCGTGGTGTTGGCAGCTCCACTAGAAGAGCATGTAAGAAGGAAAAGATTTACGTGTGATGTCCTGGGTTCA
ATTGAGGTTCAAGGTAACCGAATTAATGATGCAGCGTGCGCCCTTCATTGCCTTTGGAAAGGAGAATCTGGCGGATGGTGCGATGACCAAAAGCGGTGCAACTGTCGGGAT
TAA
>CDS_defensin_g3
ATGAAGGCAACGGCTCTTGTAATTTTACTGCTAGTAGCAGCCAATATGGTCTTAGCACATCCGCTAGAGGAGCATTTTAGGGAAAAACGTTTCTCTTGTGACATCAAGATC
ACTGCTGATGTATTTGGGGTAAAAATAACAGACACTGGTTGCAACATTCGTTGTTTCCTCGAACGCAGACCGGGTGGCACTTGTGATTCCCACAAAAAATGTCATTGTCGA
TAG
>CDS_defensin_g4
ATGAAGGCAACGGCTCTTGTAATTTTACTGCTAGTAGCAGCCAATATGGTCTTAGCACATCCGCTAGAGGAGCATTTTAGGGAAAAACGTTTCTCATGTGACATCAAGATC
ACCGCTGATGTATTTGGGGTAAAAATAACAGACACAGGTTGCAACATTCGTTGTTTCCTCGAACGCAGACCAGGTGGCACTTGTGATTCCCACAAAAAATGTCATTGTCGA
TAG
>CDS_defensin_g5
ATGAAGGCAACGGCTCTTGTAATTTTACTGCTAGTAGCAGCCAATATGGTCTTAGCACATCCGCTAGAGGAGCATTTTAGGGAAAAACGTTTCTCTTGTGACATCAAGATC
ACTGCTGATGTATTTGGGGTAAAAATAACAGACACTGGTTGCAACATTCGTTGTTTCCTCGAACGCAGACCGGGTGGCACTTGTGATTCCCACAAAAAATGTCATTGTCGA
TAG
>CDS_defensin_g6
ATGAAGGCAACTGTTGTTGTAATTTTACTGCTAGTAGCAGCCAATATGGTCTTAGCACATCCGCTAGAGGAGCATTTTAGGGAAAAACGTTTCTCATGTGACATCAAGATC
ACCGCTGATGTATTTGGGGTAAAAATAACAGACACTGGTTGCAACATTCGTTGTTTCCTCGAACGCAGACAAGGTGGCACTTGTGATTCCCACAAAAAGTGTCATTGTCGA
TAG
>CDS_defensin_g7
ATGAAGGCCGTTTTGTTGGTATTTCTCCTGGTTGTCTGCGCTGTGGTGTTGGCAGCTCCACTAGAAGAGCACATAAGAAGAAAAAGGTTTACGTGTGATGTTTTGGGATCC
GTGGACATAAAGGGTGTTCAATTAAATGATGCCCCTTGTGCCTTACATTGCCTCTGGGACGGAAAATCTGGCGGGCATTGTGATGACAGCAAAGTATGCATCTGCCGCGAT
TAA
>CDS_defensin_g8
ATGAAGGCCGTTTTGTTGGTATTTCTTCTGGTTGTTTGCGCTGTGGTGTTGGCAGCTCCATTAGAAGAGCACATAAGAAGAAAAAGGTTTACGTGCGATGTCCTGGGATCA
GTAAGCATAAAGGATGTTCAATTAAATGATGCCCCTTGTGCCTTACATTGCCTCTGGGACGGAAAATCTGGCGGGCATTGTGATGACAGCAAAGTATGCATCTGCCGTGAT
TGA
>CDS_defensin_g9
ATGAAGGCGCAGATTTTCGCTTTGGCGTTCGTCGTTTGTCTCGTCGCTGCTGTGGTTGCCATGCCAGCGCCAGAAGAACCAGAGCAACACTTGAGGGTGAGGAGGGCATCC
TGTCAAGTCTTAGGGGTTGTTGGATGCTCCGCCAATTGCTTCCGTCTTGGAAGAAAGAAGGGTGGATATTGCAACAAGAACAAAATCTGTATCTGCTACAAGTAG
>CDS_defensin_g10
ATGAAGGCGCAGATTTTCGCTTTGGCGTTCGTCGTTTGTCTCGTCGCTGCTGCGGTTGCCATGCCAGCGCCAGAAGAACCAGAGCAACACTTGAGGGTGAGGAGGGCATCC
TGTCAAGTATTAGGGGTGGTTGGATGCTCCGCCAATTGCTTCCGTCTTGGTAGAAAGAAGGGTGGATATTGCGACAAGAACCAAATCTGTATCTGCTACCAGTAG
>CDS_defensin_g11
ATGAAGGCAGTGATTTTCGTCTTCCTCGCGGCCGCCTGTGTCTTGGTTTCTGCAGCGCCTGCTCAAGAACGAGAACTTTCGCGAGTGAAGCGAGTTACATGTGATTTGTTC
AGTATCGAATTCGGAGGCATCAAAGTCAACGACGGCGCATGCGCTGTGAAGTGCATCGCCTGGGTCGGCAAAGGCTACCACGGAGGGCACTGCAAAGATGGCATCTGCACT
TGCAACAAAGGCGCAGAGCAGTGA
>CDS_defensin_g12
ATGAAGGCAGTGATTTTCGTCTTCCTCGCGGCCGCCTGTGTCTTGGTTTCAGCAGCGCCTGCTCAAGAACGAGAACTTTCGCGAGTGAAGCGAGTTACATGTGATTTGTTC
AGTATCGAATTCGGAGGCATCAAAGTCAACGACGGCGCATGCGCTGTGAAGTGCATCGCCTGGGTCGGCAAAGGCTACCACGGAGGGCACTGCAAAGATGGCATCTGCACT
TGCAACAAAGGCGCAGAGCAGTGA
>CDS_defensin_g13
ATGAAGGCTGTGATTATTGCTTTCCTACTCTTTTTGAGTGTTACATTGGGAGCTGTAGCTTTGGAGGAAAAACAAGGGCATGCTCGTGGACCACGTGATCTGACGTGCATC
GAGAAAATACCACTCGTCGGCAAAGTGAACCATGCACTGTGTGCTGCATATTGCATATCTAAAGGGAAGAAAGGCGGATTCTGCAAAGGTAGAGATTGCAACTGCCATAAC
CAATGA
>CDS_defensin_g14
ATGAAGGCTGTGATTATTGTTTTTCTACTCTTTTTGAGTGTTACATTGGGAGCTGTAGCTTTGCAGGAAAAACAAGGGCATGCTCGTGAACCACGTGATCTGACTTGCATC
GAGAAAATACCGCTCGTCGGCAAAGTGAACCATGCACTGTGTGCTGCATATTGCATATCTAAAGGGAAGAAAGGCGGATTCTGCAAAGGTAGAGATTGCAACTGCCATAAC
CAATGA
>CDS_defensin_g15
ATGAAGGTTTTCATCTTTGTATTTCTTGTCATTTGCGCTATGGCTTTAGGAATGCCAGCCAATGATCAACTTAGAAGCGAAAGAGGAGCATCCTGTGAAGTAGCAGGAGCC
CTTGGATGCTCAGCCCACTGTATTTTCCTAGGCCATCCTAAAGGAGGACATTGCGTGTCTCAAACTTGTGTCTGCTATTAA
>CDS_defensin_g16_i1
ATGAAGGTTTTCATCTTTGTATTTCTTGTCATTTGCGCTATGGCTTTAGGAATGCCAGCCAATGATCAACTTAGAAGCGAAAGAGGAGCATCCTGTGAAGTAGCAGGAGCC
CTTGGATGCTCAGCCCACTGTATTTTCCTAGGCCATCCTAAAGGAGGACATTGCGTATCTCAAACTTGTGTCTGCTATTAA
>CDS_defensin_g16_i2
ATGAAGGTTTTCATCTTTGTATTTCTTGTCATTTGCGCTATGGCTTTAGGAATGCCAGCCAATGATCAACTTAGAAGCGAAAGAGGAGCATCCTGTGAAGTAGCAGGAGCC
CTTGGATGCTCAGCCCACTGTATTTTCCTAGGCCATCCTAAAGGAGGACATTGCGTATCTCAAACTTGTGTCTGCTATTAA
>CDS_termicin_g1
ATGAAGCAGTGCACCTCCGTCCTTCTCTTCGCCGCCGCCATTCTGGTCGTCATGGCAGCCATTGTCGCCAACTTTGGCGTCGATGCCTGCGACACGAACCCTTGCTGGGCC
ACCTGCCAGGCCAAACTCACCATCTACTTCAACAGCGCCTACTGCGACGGCGACAAATGCGTCTGCGTGTCGAGGCAACCTTGA
>CDS_termicin_g2
ATGAAGCAGTGCACCTCCGTCCTTCTCTTCGCCGCCTCCATTCTGGTCGTCATGGCAGCCATTGTCGCCAACTTTGGCGTCGATGCCTGCGACACGAACCCTTGCTGGGCC
ACGTGCCAGGCCAAACTCACCATCTACTTCAACAGCGCCTACTGCGACGGCGACAAATGCGTCTGCGTGTCGAGGCAACCTTGA
>CDS_termicin_g3
ATGAAGCAGTGCACCTCCGTCCTTCTCTTCGCCGCCTCCATTCTGGTCGTCATGGCAGCCATTGTCGCCAACTTTGGCGTCGATGCCTGCGACACGAACCCTTGCTGGGCC
ACCTGCCAGGCCAAACTCACCATCTACTTCAACAGCGCCTACTGCGACGGTGACAAATGCGTCTGTGTGTCGAGGCAACCTTGA
>CDS_drosomycin_g1
ATGAAGGGATATTTGGCCATCGTAGTTCTGCTGGTTCTCACCATTGGCGCTGAGATCGCACTTGGGGACTGTTTGTCCGGAAGATACGGAGGTCCTTGTGCTGTTTGGGAC
AACGAAACCTGCCGCAGGGTGTGCAAGGAAGAAGGGCGTTCGAGTGGACACTGCAGTCCATCCCTCAAATGCTGGTGCGAGGGCTGTTGA
```

>CDS_drosomycin_g2
ATGAAGGGATATTTGGTCTTCGTAGTTCTGCTGGTTCTCACCATTGGCGCTGAGAACGCACTTGGGGACTGTTTGTCCGGAAGATACGGAGGTCCTTGTGCTGTTTGGGAC
AACGACACTTGCCGCAGGGTGTGCAAGGAAGAAGGGCGCTCGAGTGGACACTGCAGTCCATCCCTCAAATGCTGGTGCGAGGGTTGTTGA
>CDS_drosomycin_g3
ATGAAGGGATATTTGGTCTTCGTAGTTCTGCTGGTTCTCGCCATTGGCGCTGAGATCGCACTTGGGGACTGTTTGTCCGGGAGATATAAAGGTCCTTGTGCTGTTTGGGAC
AACGACACTTGCCGCAGGGTGTGCAAGGAAGAAGGGCGTTCGAGTGGACACTGCAGTCCATCCCTCAAATGCTGGTGCGAGGGTTGTTGA
>CDS_drosomycin_g4
ATGAAGGGATATTTGGCCATCGTAGTTCTGCTGGTTCTCGCCATTGGTGCTGAAATCGCACTTGGGGACTGTTTGTCCGGGAGATACGGAGGTCCTTGTGCTGTTTGGGAC
AACGACACTTGCCGCAGGGTGTGCAAGGAAGAAGGGCGCTCGAGTGGACACTGCAGTCCATCCCTCAAATGCTGGTGCGAAGGCTGTTGA
>CDS_drosomycin_g5
ATGAAGGGGTACTTGTTGTTCACCGTTCTTTTGATCCTCGCCATTGGCACCGAGATTGCTCTTGGAGATTGTCTTTCTGGAAGATATGGTGGTCCTTGTGCAGTATGGGAC
AACGAGACATGTCGGCGAGTATGCCGTGAAGAAGGACGCAGAAGTGGTCACTGCAGCGCCAGTCTCAAATGTTGGTGCGAAGGCTGTTGA
>CDS_drosomycin_g6
ATGAAGGGATATCTCATCTACACCATTCTACTCGTTCTCACCATTGGTACTACGACTGTATTATCTGATTGCAAAATATTGTCGCATCATTTTAAGGGTCCTTGTTATCCA
AGATATGTTTCGCATGGCAAGGAATGTCGCCGAAAATGTGTTCAAGAAGGTTTTATAAATGGCCGCTGCACCCCTAATCACAAATGTTATTGCAATCGCTGTTGA
>CDS_drosomycin_g7
ATGAAGGGATATCTCATCTTCACCATTCTACTCGTCCTCACCATTGGCACTGCGGTCGTGGTATCTGACTGCCCCTCAGGAAAATATAAGGGACCCTGTTTCGCTTGGAAT
AACGAGAAATGTCGCCGAATTTGTAAGGAAGAAGGTCATGTAAGTGGTCACTGTAGCGCTGGTCTCAAGTGCTGGTGTGAAGGGTGTTGA
>CDS_drosomycin_g8
ATGAAGGGATATCTCATCTTCACCGTTCTACTCGTCCTCACCATTGGCACTGCGGTCGTGTTATCTGACTGCCCCTCAGGGAAATTTAAGGGACCCTGTTTCGCATGGGAT
AACGAGAAATGTCGCCGAATTTGTAAGGAAGAAGGTCGTATAAGTGGCCACTGCAGCGCTGGTCTCAAATGTTGGTGTGAAGGGTGTTGA
>CDS_drosomycin_g9
ATGAAGGGCTACTTGTTGTTCGCCGTTCTTCTGATCCTCGCCATTGGCACCGAGATTGCTCTGGGTGATTGTCTTTCCGGAAGATACAAGGGTCCATGTGCAGTATGGGAC
AATGAAACATGCCGCCGAGTCTGTGGTGAAGAAGGACGCAGAAGTGGTCACTGCAGCCCCAGTCTCAAATGTTGGTGCGAAGGGTGTTAA
>CDS_drosomycin_g10
ATGAAGGGATATCTCATCTTCACCGTTCTACTCGTCCTCACCATTGGCACTGCGGTCGTGGTATCTGACTGCCCCTCAGGAAAATATAAGGGACCCTGTTTCGCATGGAAT
AACGAGAAATGTCGCCGAATTTGTAAGGAAGAAGGTCATATAAGTGGCCACTGCAGCGCTGGTCTCAAGTGCTGGTGTGAAGGGTGTTGA
>CDS_drosomycin_g11
ATGAAGGGATATCTCATCTTCGCCATTCTACTCATTCTCGCCATTGGCACTGCGGTAGTGTTATCTGACTGCCCTTCAGGAAAATTTAAGGGGCCCTGTTTCGCATGGAAT
AACGAAAAATGTCGCCGAATTTGTATTGAAGAAGGTCATGTAAGTGGCCACTGTAGCGCTGGTCTCAAGTGCTGGTGTGAAGGGTGTTGA
>CDS_drosomycin_g12
ATGAAGGGATATCTCATATTCACCGTTCTACTGGTTCTCACCATTGGCACTGCGGTCGTGTTATCTGACTGCCCGTCAGGAAAATTTAAGGGACCCTGTTTCGCCTGGGAT
AATGAGAAATGTCGCCGAATTTGTAAGGAAGAAGGTCGTGTAAGTGGCCACTGCAGCGCTGGTCTCAAATGTTGGTGTGAAGGGTGTTGA
>CDS_drosomycin_g13
ATGAAGGGATATCTCATCTTCACCGTTCTACTCATCCTCACCATTGGCACTGCGGTAGTGTTATCTGACTGCCCCTCAGGGAAATTTAAGGGACCCTGTTTCGCATGGGAT
AACGAGAAATGTCGCCGAATTTGTAAGGAAGAAGGTCGTGTAAGTGGCCACTGCAGCGCGGGTCTCAAGTGCTGGTGTGAAGGTTGTTGA
>CDS_Attacin-like_g1
ATGAAGGTATCCGCCATTCTATTTATTGCCCTTAGCATCGCAGTGGCTAGCACTCTGGCGAGGCCACATCACCACCCAGATCACAAAGAAGACGATCATCAGGAACAACCA
CAGGAAACTCATACTCGCGTAGAAAGGTCGCCTCAAGATGATAAGAAAGTCAACGTGCACGGGGGTATACACGAGCAGAGTGGAGTAGGTGTTGTGGCAGACGTTCATGGA
CAAGGCACAGTCTGGAAAAGCGACGATGGCAAAAGACGGGTCAATGTGGAAGGAAACTGGTCAAAGGTCCTCGATGGACCCCAGAGAGGCAAACCTCAACACAGTGCAGGT
GTCAGTTTTGATTTCGATTGGTGA
>CDS_Attacin-like_g2
ATGAAGGTCACCGCCATCTTGTTCTTAGTTCTGGCCATCGTAGTGGCTAGTGCAGTAGCAAAACCACATCATCATCCTGATCACAAGGAGGATGATCATCAGGAGCAACCA
CAGGAAAACCACAGCAGAGTTGTAAGATCCCCTCAGCCTGAAGACAAGAAAGGCAACGTGCATGGTGGAGTCCACGAAGAAAGCGGAAAAGGAGTCGTTGCTGATATTCAT
GGGAAAGGCACTGTCTGGGAGAGCAAAGATGGTAAAAGCCGAGTACAAGTAGAGGGAGACTGGTCAAAGGTCATCGATGGACCCCAGAGAGGCAAACCTCAGCATAGAGGA
GGCGTCAGTTTCGATTTTGATTTTTAA
>CDS_Attacin-like_g3A
ATGAAGGTATCCGCCATTCTATTTATTGCCCTCAGCATCGCAGTGGCTAGCACTCTGGCGAGGCCACATCACCACCCAGATCACAAAGAAGACGATCATCAGGAACAACCA
CAGGAAACTCATACTCGCGTAGAAAGATCGCCTCAAGATGACAAGAAAGGCAACGTGCACGGGGGTATACACGAGCAGAGTGGAGTAGGTGTTGTGGCAGACATTCATGGA
CAAGGCACAGTCTGGAAAAGCGACGATGGGAAAAGACGGGTCAATGTGGAAGGAAACTGGTCAAAGGTTCTCGATGGACCCCAGAGAGGCAAACCTCAACACAGTGCTGGT
GTCAGTTTTGATTTCGATTGGTAA
>CDS_Attacin-like_g3B
ATGAAGGTATCCGCCATTCTATTTATTGCCCTCAGCATCGCAGTGGCTAGCACTCTGGCGAGGCCACATCACCACCCAGATCACAAAGAAGACGATCATCAGGAACAACCA
CAGGAAACTCATACTCGCGTAGAAAGATCGCCTCAAGATGACAAGAAAGGCAACGTGCACGGGGGTATACACGAGCAGAGTGGAGTAGGTGTTGTGGCAGACATTCATGGA
CAAGGCACAGTCTGGAAAAGCGACGATGGGAAAAGACGGGTCAATGTGGAAGGAAACTGGTCAAAAGTTCTCGATGGACCCCAGAGAGGCAAACCTCAACACAGTGCTGGT
GTCAGTTTTGATTTCGATTGGTAA
>CDS_blattellicin_g1
ATGAAGATCACCGCCATTACCTTTCTTGTTATGAGCATTGCAGTGGCTAGCTCATTTGGAAGGCCACAAGGCACAGAGGAGGAAATTCAGAAGAAACTCAAGGAAACATTT
AAAGAAAATGGTGTCATGGTTTTGCTTGAAAGAATAGAACAGAGATTAATTGGCTTTGGTGCGCCTATGTTCAATTCATCAGAGGAATTATTTGGCGAAAGAAAGAAGGAA
CGTCTAGAGAAAGTCCAAGAGAGTGTTGAAACAGCTGAAGAATCAGAACAAGAACAGCAAGAACAACAAGAACAGCAACAACAGCAAGAACAACAAGAACAGCAAGAACAA
CAAGAACACCAAGAACAACAAGAACACCAAGAACAACAAGAACACCAAGAACAACAAGAACACCAAGAACACCAAGAACACCAAGAACAACCAGAACAAGAACACCAAGAA
CAACCAGAACAAGAACAGCAAGAACAACAAGAACAGCAAGAACAACAAGAACAAGAACCAGAACCAGTTCCAGCACCCCGTTTAGTTCGATCAGCACTTCCAGAAGACCAA
TCAGAAGGTATACACGCAGGTATAAATCAGCAGCCTGGCGTAGGAACAGTGGTGGATGTCAGTGGAAGAAAAAATGTTTATGAAACTGATGATGGGAGAGGTCGGGTCAAT
GTGGAGGGACAGTGGTCCAAGGTCATTGATGGACCCGGGAGAGGAAAACCTCAAGCAGGCGCTGGAATCAACTTCGAATATAAGTTTAGAAAATAG
>CDS_blattellicin_g2
ATGAAGATCACCGCCGTTACCTTTCTTGTGCTGAGCATTGCCGTGGCAAGCTCATTTGGAAGGCCACAAGCAACAGAGGAGGAAATTCAGAAGAAACTCAAGGAAACATTT
AAAGAAAATGGTTTCATGGTTTTGCTTGAAAGAATAGAACAGAGACTAATTGGATTTGGTGCGCCTATTTTCAATTCAACAGAGGAATTATTTGGCGGAAGAATGAAAGAA
CGTCTAGAGAAAGTCCAAGAGAGTGTTGAAACAGCTGAAGAATCAGAACAAGAACAGCAAGAACAACAAGAACAGCAACAACAGCAAGAACAACAAGAACAGCAAGAACAA
CAAGAACACCAAGAACAAGAACAACAAGAACACCAAGAACATCAAGAACAACCAGAACAAGAACACCAAGAACAACCAGAACAAGAACACCAAGAACAACCAGAACAAGAA
CAGCAAGAACAACAAGAACAAGAACCAGAACCAGTTCCAGCACCCCGTTTAGTTCGATCAGCACTTCCAGAAGACCAATCAGAAGGTATACACGCAGGTATAAATCAGCAG
CCTGGCGTAGGAACAGTGGTGGATGTCAGTGGAAGAAAAAATGTTTATGAAACTGATGATGGGAGAGGTCGGGTCAATGTGGAGGGACAGTGGTCCAAGGTCATTGATGGA
CCCGGGAGAGGAAAACCTCAAGCAGGCGCTGGAATCAACTTCGAATATAAGTTTAGAAAATAG
>CDS_blattellicin_g3
ATGAAGATCACCGCCATTACCTTTCTTGTTCTGAGCATTGCAGTGGCTACCTCATTTGGAAGGCCACAAGCCACAGAGGAGGAAATTCAGAAGAAACTCAAGGAAACATTT
AAAGAAAATGGTGTCATGGTTTTGCTTGAAAGAATAGAACAGAGATTAATTGGCTTTGGTGCGCCTATGTTCGATTCACCAGAGGAATTATTTGGCGAAAGAAAGAAGGAA
CGTCTAGAGAAAATCCAAGAGAGTGTTGAAACAGCTGAAGAATCAGAACAAGAACAGCAAGAACAAGAACAACAAGAACACCAAGAACAACAAGAACACCAAGAACAACAA
GAACACCAAGAACAACAAGAACACCAAGAACAACCAGAACAAGAACACCAAGAACAACCAGAACAAGAACACCAAGAACAACCAGAACAAGAACACCAAGAACAACCAGAA
CATGAACAGCAAGAACAACAAGAACAACAAGAACAGCAAGAACAACAAGAACAGCAAGAACAACAAGAACAGCATGAACAAGAACAACAAGAACAAGAACCAGAACTAGTT
CCAGCATCCCGTTTAGTACGATCAGCACTTCCAGAAGACCAATCAGAAGGTATACACGCAGGTATAAATCAGCAGCCTGGCGTAGGAACAGTGGTGGATGTCAGTGGAAGG
AAAAATGTTTATGAAACTGATGATGGGAGAGGTCGGGTCAATGTGGAGGGACAGTGGTCCAAGGTCATTGATGGACCCGGGAGAGAAAAACCTCAAGCAGGCGCTGGAATC
AACTTCGAATATAAGTTTAGAAAATAG

```
>CDS_blattellicin_g4
ATGAAGATCACCGCCATTACCTTTCTTGTTATGAGCATTGCAGTGGCTAGCTCATTTGGAAGGCCACAAACCACAGAGGAGGAAATTCAGAAGAAACTGAAGGAAACATTT
AAAGAAAATGGTTTCATGGTTTTGCTTGAAAAAATAGAACAGAGACTAATTGGCTTTGGTGCGCCTATGTTCGATTCACCAGAGGAATTATTTGGCGAAATAAAGAAGGAA
CGTCTAGAGAAAGTCCAAGAGAGTGTTGAAACAGCTGAAGAATCAGAACAAGAACACCAAGAACAACAAGAACACCAAGAACAACAAGAACACCAAGAACAACAAGAACAC
CAAGAACAACCAGAACAAGAACACCAAGAACAACCAGAACAAGAACACCAAGAACAACCAGAACATGAACAGCAAGAACAACAAGAACAACAAGAACAGCAAGAACAACAA
GAACAGCATGAACAAGAACAACAAGAACAAGAACCAGAACCAGTTCCAGCACCCCGTTTAGTTCGATCAGCACGTCCAGAAGACCAATCAGAAGGTATACACGCAGGTATA
AATCAGCAGCCTGGCGTAGGAACAGTGGTGGATGTTAGTGGAAGAAAAAATATTTATGAAACTGATGATGGGAGAGGTCGGGTCAATGTGGAGGGACAGTGGTCCAAGGTC
ATTGATGGACCCGGGAGAGGAAAACCTCAAGCAGGCGCTGGAATCAACTTCGAATATAAGTTTAGAAAATAG
```

Supplementary File 2: **B. germanica** *protein sequences*

>Defensin_g1_i1
MKSVLLVFLLVVCAVVLAAPLEEHIRRKRFTCDVLGSIEVQGNRINDAACAFHCLWKGESGGWCDDQKRCNCRD
>Defensin_g1_i2
MKSVLLVFLLVVCAVVLAAPLEEHIRRKRFTCDVLGSIEVQGNRINDAACAFHCLWKGESGGWCDDQKRCNCRD
>Defensin_g2
MKAVLLVFLLVVCAVVLAAPLEEHVRRKRFTCDVLGSIEVQGNRINDAACALHCLWKGESGGWCDDQKRCNCRD
>Defensin_g3
MKATALVILLLVAANMVLAHPLEEHFREKRFSCDIKITADVFGVKITDTGCNIRCFLERRPGGTCDSHKKCHCR
>Defensin_g4
MKATALVILLLVAANMVLAHPLEEHFREKRFSCDIKITADVFGVKITDTGCNIRCFLERRPGGTCDSHKKCHCR
>Defensin_g5
MKATALVILLLVAANMVLAHPLEEHFREKRFSCDIKITADVFGVKITDTGCNIRCFLERRPGGTCDSHKKCHCR
>Defensin_g6
MKATVVVILLLVAANMVLAHPLEEHFREKRFSCDIKITADVFGVKITDTGCNIRCFLERRQGGTCDSHKKCHCR
>Defensin_g7
MKAVLLVFLLVVCAVVLAAPLEEHIRRKRFTCDVLGSVDIKGVQLNDAPCALHCLWDGKSGGHCDDSKVCICRD
>Defensin_g8
MKAVLLVFLLVVCAVVLAAPLEEHIRRKRFTCDVLGSVSIKDVQLNDAPCALHCLWDGKSGGHCDDSKVCICRD
>Defensin_g9
MKAQIFALAFVVCLVAAVVAMPAPEEPEQHLRVRRASCQVLGVVGCSANCFRLGRKKGGYCNKNKICICYK
>Defensin_g10
MKAQIFALAFVVCLVAAAVAMPAPEEPEQHLRVRRASCQVLGVVGCSANCFRLGRKKGGYCDKNQICICYQ
>Defensin_g11
MKAVIFVFLAAACVLVSAAPAQERELSRVKRVTCDLFSIEFGGIKVNDGACAVKCIAWVGKGYHGGHCKDGICTCNKGAEQ
>Defensin_g12
MKAVIFVFLAAACVLVSAAPAQERELSRVKRVTCDLFSIEFGGIKVNDGACAVKCIAWVGKGYHGGHCKDGICTCNKGAEQ
>Defensin_g13
MKAVIIAFLLFLSVTLGAVALEEKQGHARGPRDLTCIEKIPLVGKVNHALCAAYCISKGKKGGFCKGRDCNCHNQ
>Defensin_g14
MKAVIIVFLLFLSVTLGAVALQEKQGHAREPRDLTCIEKIPLVGKVNHALCAAYCISKGKKGGFCKGRDCNCHNQ
>Defensin_g15
MKVFIFVFLVICAMALGMPANDQLRSERGASCEVAGALGCSAHCIFLGHPKGGHCVSQTCVCY
>Defensin_g16_i1
MKVFIFVFLVICAMALGMPANDQLRSERGASCEVAGALGCSAHCIFLGHPKGGHCVSQTCVCY
>Defensin_g16_i2
MKVFIFVFLVICAMALGMPANDQLRSERGASCEVAGALGCSAHCIFLGHPKGGHCVSQTCVCY
>Termicin_g1
MKQCTSVLLFAAAILVVMAAIVANFGVDACDTNPCWATCQAKLTIYFNSAYCDGDKCVCVSRQP
>Termicin_g2
MKQCTSVLLFAASILVVMAAIVANFGVDACDTNPCWATCQAKLTIYFNSAYCDGDKCVCVSRQP
>Termicin_g3
MKQCTSVLLFAASILVVMAAIVANFGVDACDTNPCWATCQAKLTIYFNSAYCDGDKCVCVSRQP
>Drosomycin_g1
MKGYLAIVVLLVLTIGAEIALGDCLSGRYGGPCAVWDNETCRRVCKEEGRSSGHCSPSLKCWCEGC
>Drosomycin_g2
MKGYLVFVVLLVLTIGAENALGDCLSGRYGGPCAVWDNDTCRRVCKEEGRSSGHCSPSLKCWCEGC
>Drosomycin_g3
MKGYLVFVVLLVLAIGAEIALGDCLSGRYKGPCAVWDNDTCRRVCKEEGRSSGHCSPSLKCWCEGC
>Drosomycin_g4
MKGYLAIVVLLVLAIGAEIALGDCLSGRYGGPCAVWDNDTCRRVCKEEGRSSGHCSPSLKCWCEGC
>Drosomycin_g5
MKGYLLFTVLLILAIGTEIALGDCLSGRYGGPCAVWDNETCRRVCREEGRRSGHCSASLKCWCEGC
>Drosomycin_g6
MKGYLIYTILLVLTIGTTTVLSDCKILSHHFKGPCYPRYVSHGKECRRKCVQEGFINGRCTPNHKCYCNRC
>Drosomycin_g7
MKGYLIFTILLVLTIGTAVVVSDCPSGKYKGPCFAWNNEKCRRICKEEGHVSGHCSAGLKCWCEGC
>Drosomycin_g8
MKGYLIFTVLLVLTIGTAVVLSDCPSGKFKGPCFAWDNEKCRRICKEEGRISGHCSAGLKCWCEGC
>Drosomycin_g9
MKGYLLFAVLLILAIGTEIALGDCLSGRYKGPCAVWDNETCRRVCGEEGRRSGHCSPSLKCWCEGC
>Drosomycin_g10
MKGYLIFTVLLVLTIGTAVVVSDCPSGKYKGPCFAWNNEKCRRICKEEGHISGHCSAGLKCWCEGC
>Drosomycin_g11
MKGYLIFAILLILAIGTAVVLSDCPSGKFKGPCFAWNNEKCRRICIEEGHVSGHCSAGLKCWCEGC
>Drosomycin_g12
MKGYLIFTVLLVLTIGTAVVLSDCPSGKFKGPCFAWDNEKCRRICKEEGRVSGHCSAGLKCWCEGC
>Drosomycin_g13
MKGYLIFTVLLILTIGTAVVLSDCPSGKFKGPCFAWDNEKCRRICKEEGRVSGHCSAGLKCWCEGC
>Attacin-like_g1
MKVSAILFIALSIAVASTLARPHHHPDHKEDDHQEQPQETHTRVERSPQDDKKVNVHGGIHEQSGVGVVADVHGQGTVWKSDDGKRRVNVEGNWSKVLDGPQRGKPQHSAG
VSFDFDW
>Attacin-like_g2
MKVTAILFLVLAIVVASAVAKPHHHPDHKEDDHQEQPQENHSRVVRSPQPEDKKGNVHGGVHEESGKGVVADIHGKGTVWESKDGKSRVQVEGDWSKVIDGPQRGKPQHRG
GVSFDFDF
>Attacin-like_g3A
MKVSAILFIALSIAVASTLARPHHHPDHKEDDHQEQPQETHTRVERSPQDDKKGNVHGGIHEQSGVGVVADIHGQGTVWKSDDGKRRVNVEGNWSKVLDGPQRGKPQHSAG
VSFDFDW
>Attacin-like_g3B

MKVSAILFIALSIAVASTLARPHHHPDHKEDDHQEQPQETHTRVERSPQDDKKGNVHGGIHEQSGVGVVADIHGQGTVWKSDDGKRRVNVEGNWSKVLDGPQRGKPQHSAG
VSFDFDW
>Blattellicin_g1
MKITAITFLVMSIAVASSFGRPQGTEEEIQKKLKETFKENGVMVLLERIEQRLIGFGAPMFNSSEELFGERKKERLEKVQESVETAEESEQEQQEQQEQQQQQEQQEQQEQ
QEHQEQQEHQEQQEHQEQQEHQEHQEHQEQPEQEHQEQPEQEQQEQQEQQEQQEQEPEPVPAPRLVRSALPEDQSEGIHAGINQQPGVGTVVDVSGRKNVYETDDGRGRVN
VEGQWSKVIDGPGRGKPQAGAGINFEYKFRK
>Blattellicin_g2
MKITAVTFLVLSIAVASSFGRPQATEEEIQKKLKETFKENGFMVLLERIEQRLIGFGAPIFNSTEELFGGRMKERLEKVQESVETAEESEQEQQEQQEQQQQQEQQEQQEQ
QEHQEQEQQEHQEHQEQPEQEHQEQPEQEHQEQPEQEQQEQQEQEPEPVPAPRLVRSALPEDQSEGIHAGINQQPGVGTVVDVSGRKNVYETDDGRGRVNVEGQWSKVIDG
PGRGKPQAGAGINFEYKFRK
>Blattellicin_g3
MKITAITFLVLSIAVATSFGRPQATEEEIQKKLKETFKENGVMVLLERIEQRLIGFGAPMFDSPEELFGERKKERLEKIQESVETAEESEQEQQEQEQQEHQEQQEHQEQQ
EHQEQQEHQEQPEQEHQEQPEQEHQEQPEQEHQEQPEHEQQEQQEQQEQQEQQEQQEQQEQHEQEQQEQEPELVPASRLVRSALPEDQSEGIHAGINQQPGVGTVVDVSGR
KNVYETDDGRGRVNVEGQWSKVIDGPGREKPQAGAGINFEYKFRK
>Blattellicin_g4
MKITAITFLVMSIAVASSFGRPQTTEEEIQKKLKETFKENGFMVLLEKIEQRLIGFGAPMFDSPEELFGEIKKERLEKVQESVETAEESEQEHQEQQEHQEQQEHQEQQEH
QEQPEQEHQEQPEQEHQEQPEHEQQEQQEQQEQQEQQEQHEQEQQEQEPEPVPAPRLVRSARPEDQSEGIHAGINQQPGVGTVVDVSGRKNIYETDDGRGRVNVEGQWSKV
IDGPGRGKPQAGAGINFEYKFRK

Supplementary Table 1. Genes encoding proteins with antimicrobial peptide domains annotated in the genome of *Blattella germanica* (GCA_003018175.1).

No.	locus_tag	PFAM code	PFAM_name	product	partial	protein_id	Coding Exons	Scaffold Accession No.
1	C0J52_22336	PF01097	Defensin_2	Tenecin-1		PSN36807.1	2	PYGN01001185.1
2	C0J52_22338	PF01097	Defensin_2	Tenecin-1		PSN36809.1	2	PYGN01001185.1
3	C0J52_22339	PF01097	Defensin_2	Defense protein 6		PSN36808.1	2	PYGN01001185.1
4	C0J52_22340	PF01097	Defensin_2	Tenecin-1		PSN36810.1	2	PYGN01001185.1
5	C0J52_24004	PF01097	Defensin_2	Tenecin-1		PSN30606.1	2	PYGN01002380.1
6	C0J52_24005	PF01097	Defensin_2	Phormicin		PSN30605.1	2	PYGN01002380.1
7	C0J52_24006	PF01097	Defensin_2	Tenecin-1		PSN30608.1	2	PYGN01002380.1
8	C0J52_27569	PF01097	Defensin_2	Tenecin-1		PSN29532.1	2	PYGN01003429.1
9	C0J52_20459	PF01097	Defensin_2	hypothetical protein	**partial=5'**	PSN47761.1	2	PYGN01000358.1
10	C0J52_20460	PF01097	Defensin_2	hypothetical protein	**partial=5'**	PSN47759.1	2	PYGN01000358.1
11	C0J52_03170	PF00304	Gamma-thionin	Drosomycin		PSN55662.1	1	PYGN01000062.1
12	C0J52_03171	PF00304	Gamma-thionin	Drosomycin		PSN55663.1	1	PYGN01000062.1
13	C0J52_12810	PF00304	Gamma-thionin	Drosomycin		PSN34060.1	1	PYGN01001559.1
14	C0J52_12811	PF00304	Gamma-thionin	Drosomycin		PSN34059.1	1	PYGN01001559.1
15	C0J52_12812	PF00304	Gamma-thionin	Drosomycin		PSN34058.1	1	PYGN01001559.1
16	C0J52_12813	PF00304	Gamma-thionin	Drosomycin		PSN34057.1	1	PYGN01001559.1
17	C0J52_23105	PF00304	Gamma-thionin	Drosomycin		PSN31135.1	1	PYGN01002215.1
18	C0J52_23106	PF00304	Gamma-thionin	Drosomycin		PSN31132.1	1	PYGN01002215.1
19	C0J52_23107	PF00304	Gamma-thionin	Drosomycin		PSN31136.1	1	PYGN01002215.1
20	C0J52_23108	PF00304	Gamma-thionin	Drosomycin		PSN31134.1	1	PYGN01002215.1
21	C0J52_00758	PF11415	Toxin_37	hypothetical protein		PSN51483.1	3	PYGN01000196.1
22	C0J52_26761	PF11415	Toxin_37	hypothetical protein		PSN29824.1	3	PYGN01002934.1
23	C0J52_26762	PF11415	Toxin_37	hypothetical protein		PSN29822.1	3	PYGN01002934.1
24	C0J52_26498	PF03769	Attacin_C	hypothetical protein		PSN32650.1	10	PYGN01001824.1

Supplementary Table 2. Genes encoding *B. germanica* AMPs ordered by scaffold position. The * indicates that a partial sequence of the transcript is detected in the genome. See supplementary Tables 3, 4, 5 and 6 for additional information.

No.	Scaffold	Gene	Equivalent CDS Locus_tag	Product	CDS length	Protein length	CDS_location	mRNA_location
1	PYGN01000062	*drosomycin_g2*	C0J52_03170	Drosomycin	201	66	complement(134469..134669)	complement(134414..134710)
1	PYGN01000062	*drosomycin_g3*	C0J52_03171	Drosomycin	201	66	141391..141591	141342..141730
2	PYGN01000196	*termicin_g1*	C0J52_00758	Termicin	195	64	1073896..1073977,1074070..1074165,1074283..1074299	1073759..1073977,1074070..1074165,1074283..1074496
3	PYGN01000358	*defensin_g15*	C0J52_20459	Defensin-like	192	63	1126699..1126762,1128631..1128758	1126679..1126762,1128631..1129086
3	PYGN01000358	*defensin_g16*	C0J52_20460	Defensin-like	192	63	1143127..1143190,1143402..1143529	1143107..1143190,1143402..1143533
4	PYGN01001185	*defensin_g8*	C0J52_22336	Tenecin-1	225	74	23826..23892,25077..25234	23788..23892,25077..25236,25768..25896
4	PYGN01001185	*defensin_g5*	C0J52_22338	Tenecin-1	225	74	42981..43050,44095..44249	42905..43050,44095..44348
4	PYGN01001185	*defensin_g10*		Phormicin	216	71	54069..54200 *	54067..54477 *
4	PYGN01001185	*defensin_g13*	C0J52_22339	Defense_Protein_6	228	75	56543..56618,57654..57805	56525..56618,57654..57912
4	PYGN01001185	*defensin_g14*		Defense_Protein_6	228	75	59448..59523,60558..60568 *	59448..59523,60558..60569 *
4	PYGN01001185	*defensin_g12*	C0J52_22340	Tenecin-1	246	81	62286..62358,64095..64267	61977..62358,64095..64380
5	PYGN01001559	*drosomycin_g5*	C0J52_12810	Drosomycin	201	66	24475..24675	24292..25288
5	PYGN01001559	*drosomycin_g6*		Drosomycin	216	71	complement(26896..27111)	complement(26881..27333)
5	PYGN01001559	*drosomycin_g7*	C0J52_12811	Drosomycin	201	66	complement(28937..29137)	complement(28937..29137)
5	PYGN01001559	*drosomycin_g8*	C0J52_12812	Drosomycin	201	66	32609..32809	32609..32809
5	PYGN01001559	*drosomycin_g9*	C0J52_12813	Drosomycin	201	66	46501..46701	46501..46701
6	PYGN01001824	*attacin-like_g1*	C0J52_26498	Attacin-like	357	118	complement(27121..27363,31556..31669)	complement(27040..27363,31556..31679,33633..33670)
6	PYGN01001824	*attacin-like_g2*	C0J52_26498	Attacin-like	360	119	complement(46993..47238,51839..51952)	complement(46937..47238,51839..51964,53516..53577)
6	PYGN01001824	*blattellicin_g1*	C0J52_26498	Blattellicin	762	253	complement(59302..59583_gap,gap_61539..61679,62299..62451)	
6	PYGN01001824	*blattellicin_g4*	C0J52_26498	Blattellicin	738	245	complement(64600..64752)*	
6	PYGN01001824	*blattellicin_g2*	C0J52_26498	Blattellicin	729	242	complement(74091..74243)*	
7	PYGN01002215	*drosomycin_g10*	C0J52_23105	Drosomycin	201	66	complement(23648..23848)	complement(23648..23848)
7	PYGN01002215	*drosomycin_g11*	C0J52_23106	Drosomycin	201	66	complement(27214..27414)	complement(27214..27414)
7	PYGN01002215	*drosomycin_g12*	C0J52_23107	Drosomycin	201	66	32000..32200	32000..32200
7	PYGN01002215	*drosomycin_g13*	C0J52_23108	Drosomycin	201	66	33789..33989	33789..33989
8	PYGN01002380	*defensin_g2*	C0J52_24001	Tenecin-1	225	74	35554..35620,36253..36410	35542..35620,36253..37002
8	PYGN01002380	*defensin_g7*		Tenecin-1	225	74	40436..40502,43160..43317	40436..40502,43160..43319,43857..43985
8	PYGN01002380	*defensin_g6*	C0J52_24004	Tenecin-1	225	74	61809..61878,63258..63412	61730..61878,63258..63511
8	PYGN01002380	*defensin_g9*	C0J52_24005	Phormicin	216	71	70553..70634,71926..72059	70532..70633,71926..72292
8	PYGN01002380	*defensin_g11*	C0J52_24006	Tenecin-1	246	81	77883..77955,79479..79651	77549..77955,79479..79762
9	PYGN01002934	*termicin_g2*	C0J52_26761	Termicin	195	64	complement(3047..3063,3175..3270,3359..3440)	complement(2817..3063,3175..3270,3359..3579)
9	PYGN01002934	*termicin_g3*	C0J52_26762	Termicin	195	64	5727..5808,5897..5992,6086..6102	5587..5808,5897..5992,6086..6350
10	PYGN01003429	*defensin_g4*	C0J52_27569	Tenecin-1	225	74	complement(10961..11115,12487..12556)	complement(10883..11115,12487..12592)
10	PYGN01003429	*defensin_g3*		Tenecin-1	225	74	complement(9202..9356,10401..10470)	complement(9130..9356,10401..10485) *
Un	Unplaced	*defensin_g1_*		Tenecin-1	225	74	Not in genome project	Not in genome project
Un	Unplaced	*drosomycin_g1*		Drosomycin	201	66	Not in genome project	Not in genome project
Un	Unplaced	*drosomycin_g4*		Drosomycin	201	66	Not in genome project	Not in genome project
Un	Unplaced	*attacin-like_g3*		Attacin-like	357	118	Not in genome project	Not in genome project
Un	Unplaced	*blattellicin_g3*		Blattellicin	804	267	Not in genome project	Not in genome project

Supplementary Table 3. *B. germanica* defensin AMP genes. TFM (transcripts per million transcripts). Hits CDS 41-190 (%) means a comparative level of transcription among defensin genes (percentage of number of hits for a concrete defensin gene/ summation of hits for all defensin genes). The * means that the 150 nucleotides (CDS 41-190) of *defensin_g3* and *defensin_g5* are 100% identical. Joint cells imply that it was not possible to determine the level of transcription of 2 or more defensin genes due to either TRINITY joint assembly or the 100% identity of the analyzed CDS sequences.

Genes	Equivalent CDS Locus_tag	Scaffold	SRR678710_assembly gene id	TPM	Hits CDS 41-190 (%)	Product	CDS length	Protein length	CDS_location	mRNA_location
defensin_g1_i1		Unplaced	TRINITY_DN1123_c0_g1	2.84	7.7	Tenecin-1	225	74	Not in genome project	Not in genome project
defensin_g1_i2		Unplaced	TRINITY_DN1123_c0_g2	58.66		Tenecin-1	225	74	Not in genome project	Not in genome project
defensin_g2	C0J52_24001	PYGN01002380	TRINITY_DN1123_c0_g3	20.52	7.8	Tenecin-1	225	74	35554..35620,36253..36410	35542..35620,36253..37002
defensin_g3		PYGN01003429	TRINITY_DN13842_c0_g1	323.64	14.6*	Tenecin-1	225	74	complement(9202..9356,10401..10470)	complement(9130..9356,10401..10485) (incomplete)
defensin_g4	C0J52_27569	PYGN01003429	TRINITY_DN13842_c0_g1		0.8	Tenecin-1	225	74	complement(10961..11115,12487..12556)	complement(10883..11115,12487..12592)
defensin_g5	C0J52_22338	PYGN01001185	TRINITY_DN13842_c0_g1		14.6*	Tenecin-1	225	74	42981..43050,44095..44249	42905..43050,44095..44348
defensin_g6	C0J52_24004	PYGN01002380	TRINITY_DN13842_c0_g1		0.5	Tenecin-1	225	74	61809..61878,63258..63412	61730..61878,63258..63511
defensin_g7		PYGN01002380	TRINITY_DN23519_c0_g1	24.70	2.3	Tenecin-1	225	74	40436..40502,43160..43317	40436..40502,43160..43319,43857..43985
defensin_g8	C0J52_22336	PYGN01001185	TRINITY_DN23519_c0_g3	24.70	2.3	Tenecin-1	225	74	23826..23892,25077..25234	23788..23892,25077..25236,25768..25896
defensin_g9	C0J52_24005	PYGN01002380	TRINITY_DN113_c0_g1	63.53	12.2	Phormicin	216	71	70553..70634,71926..72059	70532..70633,71926..72292
defensin_g10		PYGN01001185	TRINITY_DN113_c0_g2	39.55	13.2	Phormicin	216	71	54069..54200 (incomplete)	54067..54477 (incomplete)
defensin_g11	C0J52_24006	PYGN01002380	TRINITY_DN26506_c0_g1	22.10	1.4	Tenecin-1	246	81	77883..77955,79479..79651	77549..77955,79479..79762
defensin_g12	C0J52_22340	PYGN01001185	TRINITY_DN26506_c0_g1		0	Tenecin-1	246	81	62286..62358,64095..64267	61977..62358,64095..64380
defensin_g13	C0J52_22339	PYGN01001185	TRINITY_DN56319_c0_g2	0.00	0.2	Defense_Protein_6	228	75	56543..56618,57654..57805	56525..56618,57654..57912
defensin_g14		PYGN01001185	TRINITY_DN56319_c0_g1	0.00	0	Defense_Protein_6	228	75	59448..59523,60558..60568 (incomplete)	59448..59523,60558..60569 (incomplete)
defensin_g15	C0J52_20459	PYGN01000358	TRINITY_DN52201_c0_g1	7.36	12.9	Defensin-like	192	63	1126699..1126762,1128631..1128758	1126679..1126762,1128631..1129086
defensin_g16_i1	C0J52_20460	PYGN01000358	TRINITY_DN526_c0_g1	45.76	24.4	Defensin-like	192	63	1143127..1143190,1143402..1143529	1143107..1143190,1143402..1143533
defensin_g16_i2	C0J52_20460	PYGN01000358	TRINITY_DN526_c0_g2	25.9		Defensin-like	192	63	1143127..1143190,1143402..1143529	1143107..1143190,1143402..1144406

Supplementary Table 4. Number of transcripts with similarity to *B. germanica* AMPs in Blattodea. The number of transcripts with similarity to *B. germanica* Defensins, Termicins, Drosomycins and Attacins were screened in 44 TSA projects and the annotated mRNAs of *Zootermopsis nevadensis*. TBLASTN searches (e-value = 0.001) with 17 selected *B. germanica* AMP proteins as queries (see Supplementary Table 8, and Figure 5) were performed. The same TSA projects plus *Z. nevadensis* used to produce a time-calibrated phylogeny of Blattodea (Evangelista, D.A. et al. 2019. Proc. R. Soc. B286: 20182076) were analysed. Alternation of grey highlighting in taxonomic group columns indicates the clades described in the time-calibrated phylogeny of Blattodea (Figure 1 from Evangelista et al. 2019). The absence of transcript hits from an AMP type in a Blattodea species is highlighted in pink. The numbers of hits for each of the four types of AMPs in a concrete species are the intersection of the blast results of the same type of AMPs.

	Order	Group 1	Group 2	Group 3	Genus	Species epithet	Defensins mRNAs	Termicins mRNAs	Drosomycins mRNAs	Attacins mRNAs	TSA Accession
1	Blattodea	Blaberoidea	"Ectobiidae"	Blattellinae	*Asiablatta*	*kyotensis*	5	2	2	2	GDWW00000000
2	Blattodea	Blaberoidea	"Ectobiidae"	Blattellinae	*Blattella*	*germanica*	2	1	2	3	GDCR00000000
3	Blattodea	Blaberoidea	"Ectobiidae"	Blattellinae	*Episymploce*	*sundaica*	9	3	5	3	GDYC00000000
4	Blattodea	Blaberoidea	"Ectobiidae"	Blattellinae	*Ischnoptera*	*deropeltiformis*	4	0	1	1	GDEC00000000
5	Blattodea	Blaberoidea	"Ectobiidae"	Blattellinae	*Loboptera*	*decipiens*	3	1	9	2	GDYK00000000
6	Blattodea	Blaberoidea	"Ectobiidae"	Blattellinae	*Lobopterella*	*dimidiatipes*	8	2	2	1	GDZZ00000000
7	Blattodea	Blaberoidea	"Ectobiidae"	Blattellinae	*Paratemnopteryx*	*couloniana*	8	0	0	1	GDZI00000000
8	Blattodea	Blaberoidea	"Ectobiidae"	Blattellinae	*Symploce*	*sp. AD-2014*	4	5	4	2	GDCG00000000
9	Blattodea	Blaberoidea	"Ectobiidae"	Nyctiborinae	*Nyctibora*	*sp. AD-2015*	5	1	1	0	GDZE00000000
10	Blattodea	Blaberoidea	Blaberidae	Blaberinae	*Blaberus*	*atropos*	2	1	2	1	GAYD00000000
11	Blattodea	Blaberoidea	Blaberidae	Diplopterinae	*Diploptera*	*punctata*	3	1	0	1	GDYG00000000
12	Blattodea	Blaberoidea	Blaberidae	Gyninae	*Gyna*	*lurida*	3	1	0	1	GDZJ00000000
13	Blattodea	Blaberoidea	Blaberidae	Oxyhaloinae	*Nauphoeta*	*cinerea*	8	2	4	1	GDCI00000000
14	Blattodea	Blaberoidea	Blaberidae	Oxyhaloinae	*Princisia*	*vanwaerebeki*	1	0	2	1	GDXG00000000
15	Blattodea	Blaberoidea	Blaberidae	Panchlorinae	*Panchlora*	*nivea*	2	1	3	2	GDWQ00000000
16	Blattodea	Blaberoidea	Blaberidae	Zetoborinae	*Schultesia*	*lampyridiformis*	1	0	4	1	GCPR00000000
17	Blattodea	Blaberoidea	"Ectobiidae"	-	*Anallacta*	*methanoides*	3	0	0	1	GEAA00000000
18	Blattodea	Blaberoidea	"Ectobiidae"	Pseudophyllodromiinae	*Balta*	*vilis*	3	3	3	0	GDYJ00000000
19	Blattodea	Blaberoidea	"Ectobiidae"	Pseudophyllodromiinae	*Cariblatta*	*sp. AD-2015*	2	2	0	1	GDZD00000000
20	Blattodea	Blaberoidea	"Ectobiidae"	Pseudophyllodromiinae	*Ellipsidion*	*sp. AD-2015*	3	0	1	1	GDYE00000000
21	Blattodea	Blaberoidea	"Ectobiidae"	Pseudophyllodromiinae	*Euthlastoblatta*	*diaphana*	3	1	1	1	GDZY00000000
22	Blattodea	Blaberoidea	"Ectobiidae"	Pseudophyllodromiinae	*Sundablatta*	*sexpunctata*	4	0	1	1	GDCJ00000000
23	Blattodea	Blaberoidea	"Ectobiidae"	Pseudophyllodromiinae	*Supella*	*longipalpa*	3	2	1	1	GDWU00000000
24	Blattodea	Blaberoidea	"Ectobiidae"	Ectobiinae	*Ectobius*	*sylvestris*	4	0	4	0	GDYP00000000
25	Blattodea	Solumblattodea	Isoptera	Kalotermitidae	*Incisitermes*	*marginipennis*	1	2	0	1	GDBO00000000
26	Blattodea	Solumblattodea	Isoptera	Mastotermitidae	*Mastotermes*	*darwiniensis*	1	2	0	3	GAZE00000000
27	Blattodea	Solumblattodea	Isoptera	Rhinotermitidae	*Prorhinotermes*	*simplex*	3	3	0	1	GASE00000000
28	Blattodea	Solumblattodea	Isoptera	Rhinotermitidae	*Reticulitermes*	*santonensis*	1	2	0	0	GDVA00000000
29	Blattodea	Solumblattodea	Isoptera	Termitidae / Termitinae	*Coptotermes*	*sp. AD-2015*	4	3	0	0	GDUG00000000
30	Blattodea	Solumblattodea	Isoptera	Termopsidae	*Zootermopsis*	*nevadensis*	0	0	0	1	Annotated RNAs
31	Blattodea	Solumblattodea	Cryptocercidae	Cryptocercinae	*Cryptocercus*	*wrighti*	1	2	0	2	GAZN00000000
32	Blattodea	Solumblattodea	Lamproblattidae	Lamproblattinae	*Lamproblatta*	*albipalpus*	1	1	3	3	GCPS00000000
33	Blattodea	Solumblattodea	Blattidae	Archiblattinae	*Catara*	*rugosicollis*	3	2	3	1	GDCA00000000
34	Blattodea	Solumblattodea	Blattidae	Blattinae	*Deropeltis*	*erythrocephala*	1	0	1	1	GDZH00000000
35	Blattodea	Solumblattodea	Blattidae	Blattinae	*Periplaneta*	*americana*	4	0	2	1	GAWS00000000
36	Blattodea	Solumblattodea	Blattidae	Blattinae	*Shelfordella*	*lateralis*	1	0	3	1	GDXP00000000
37	Blattodea	Solumblattodea	Blattidae	Polyzosteriinae	*Eurycotis*	*floridana*	3	1	0	0	GDYS00000000
38	Blattodea	Solumblattodea	Blattidae	Polyzosteriinae	*Methana*	*parva*	4	1	0	2	GDWT00000000
39	Blattodea	Solumblattodea	Tryonicidae	Tryonicinae	*Tryonicus*	*parvus*	1	1	0	2	GDWV00000000
40	Blattodea	Solumblattodea	Corydioidea	Corydiinae	*Ergaula*	*capucina*	2	0	0	1	GDBS00000000
41	Blattodea	Solumblattodea	Corydioidea	Corydiinae	*Eucorydia*	*yasumatsui*	1	0	0	0	GDZF00000000
42	Blattodea	Solumblattodea	Corydioidea	Corydiinae	*Polyphaga*	*aegyptiaca*	3	1	0	0	GDWJ00000000
43	Blattodea	Solumblattodea	Corydioidea	Corydiinae	*Therea*	*bernhardti*	4	0	0	1	GDVQ00000000
44	Blattodea	Solumblattodea	Corydioidea	Tiviinae	*Tivia*	*sp. AD-2015*	3	1	0	0	GDYD00000000
45	Blattodea	Solumblattodea	Corydioidea	Nocticolinae	*Nocticola*	*sp. AD-2015*	3	0	0	0	GDYB00000000

Supplementary Table 5. *B. germanica* termicin AMP genes. TFM (transcripts per million transcripts). Hits CDS 31-180 (%) means a comparative level of transcription among termicin genes (percentage of number of hits for a concrete termicin gene/ summation of hits for all termicin genes).

Genes	Equivalent CDS Locus_tag	Scaffold	SRR678710_assembly gene id	TPM	hits CDS 31-180 (%)	Product	CDS length	Protein length	CDS_location	mRNA_location
termicin_g1	C0J52_00758	PYGN01000196	TRINITY_DN10017_c0_g1	9.21	0	Termicin	195	64	1073896..1073977,1074070..1074165,1074283..1074299	1073759..1073977,1074070..1074165,1074283..1074496
termicin_g2	C0J52_26761	PYGN01002934			42.9	Termicin	195	64	complement(3047..3063,3175..3270,3359..3440)	complement(2817..3063,3175..3270,3359..3579)
termicin_g3	C0J52_26762	PYGN01002934	TRINITY_DN10017_c0_g2	46.06	57.1	Termicin	195	64	5727..5808,5897..5992,6086..6102	5587..5808,5897..5992,6086..6350

Supplementary Table 6. *B. germanica* drosomycin AMP genes. TFM (transcripts per million transcripts). Hits CDS 1-150 (%) means a comparative level of transcription among drosomycin genes (percentage of number of hits for a concrete drosomycin gene/ summation of hits for all drosomycin genes).

Genes	Equivalent CDS Locus_tag	Scaffold	SRR678710_assembly id	TPM	hits CDS 1-150 (%)	Product	CDS length	Protein length	CDS_location	mRNA_location	Notes comparing with genome sequence or annotation
drosomycin_g1	Not in genome	Unplaced	TRINITY_DN37069_c0_g3_i1	10.36	1.4	Drosomycin	201	66			
drosomycin_g2	C0J52_03170	PYGN01000062	TRINITY_DN37069_c0_g4_i1	124.74	2.7	Drosomycin	201	66	complement(134469..134669)	complement(134414..134710)	1 nonsynonymous nt difference in CDS and 1 difference in UTR. CDS length modified
drosomycin_g3	C0J52_03171	PYGN01000062	TRINITY_DN37069_c0_g1_i1	71.57	8.9	Drosomycin	201	66	141391..141591	141342..141730	
drosomycin_g4	Not in genome	Unplaced	TRINITY_DN37069_c0_g6_i1	2.59	0.5	Drosomycin	201	66			
drosomycin_g5	C0J52_12810	PYGN01001559	TRINITY_DN2347_c0_g1_i1	156.82	86.1	Drosomycin	201	66	24475..24675	24292..25288	several nt differences and indels in the UTRs
drosomycin_g6		PYGN01001559	TRINITY_DN13624_c0_g1_i1	4.57	0.4	Drosomycin	216	71	complement(26896..27111)	complement(26881..27333)	2 nt differences and 1 indel in the UTRs and 1 nonsynonymous difference in the CDS are observed
drosomycin_g7	C0J52_12811	PYGN01001559		nd	0	Drosomycin	201	66	complement(28937..29137)	complement(28937..29137)	
drosomycin_g8	C0J52_12812	PYGN01001559		nd	0	Drosomycin	201	66	32609..32809	32609..32809	
drosomycin_g9	C0J52_12813	PYGN01001559		nd	0	Drosomycin	201	66	46501..46701	46501..46701	
drosomycin_g10	C0J52_23105	PYGN01002215		nd	0	Drosomycin	201	66	complement(23648..23848)	complement(23648..23848)	
drosomycin_g11	C0J52_23106	PYGN01002215		nd	0	Drosomycin	201	66	complement(27214..27414)	complement(27214..27414)	
drosomycin_g12	C0J52_23107	PYGN01002215		nd	0	Drosomycin	201	66	32000..32200	32000..32200	
drosomycin_g13	C0J52_23108	PYGN01002215		nd	0	Drosomycin	201	66	33789..33989	33789..33989	

Supplementary Table 7. *B. germanica* attacin-like and blattellicin AMP genes. TFM (transcripts per million transcripts). Hits CDS 1-150 (%) means a comparative level of transcription among drosomycin genes (percentage of number of hits for a concrete gene/ summation of hits for all genes). Only partial sequences of the mRNA are detected in the assembled genome (*).

Genes	Equivalent CDS Locus_tag (segment of..)	Scaffold	SRR678710_assembly id	TPM	hits CDS 1-150 (%)	Product	CDS length	Protein length	CDS_location	mRNA_location	Notes comparing with genome sequence or annotation
attacin-like_g1	C0J52_26498	PYGN01001824	TRINITY_DN950_c0_g1_i1_len=786	10.37	6.9	Attacin-like	357	118	complement(27121..27363,31556..31669)	complement(27040..27363,31556..31679,33633..33670)	
attacin-like_g2	C0J52_26498	PYGN01001824	TRINITY_DN54826_c0_g1_i1_len=490	8.17	1.6	Attacin-like	360	119	complement(46993..47238,51839..51952)	complement(46937..47238,51839..51964,53516..53577)	3 dif in mRNA
attacin-like_g3A	Not in genome	Unplaced	TRINITY_DN950_c0_g2_i1_len=777	4.41	7.3	Attacin-like	357	118			
attacin-like_g3B	Not in genome	Unplaced				Attacin-like	357	118			
blattellicin_g1	C0J52_26498	PYGN01001824	several incomplete transcripts		13	Blattellicin	762	253	complement(59302..59583_gap,gap_61539..61679,62299..62451)		second and third exon limited by gaps
blattellicin_g2	C0J52_26498	PYGN01001824	several incomplete transcripts		3.2	Blattellicin	729	242	complement(74091..74243)*		*only 1st CDS exon identified
blattellicin_g3		Unplaced	several incomplete transcripts		9.7	Blattellicin	804	267			
blattellicin_g4	C0J52_26498	PYGN01001824	several incomplete transcripts		58.2	Blattellicin	738	245	complement(64600..64752)*		*only 1st CDS exon identified

Supplementary Table 8. Abundance of transcripts for 17 selected AMP genes in Sequence Read (SR) experiments corresponding to whole bodies from diverse developmental stages of *B. germanica* (1-28), whole body mixtures of developmental stages or sexes (29-33) and parts of the body or tissues (34-55). Values were estimated as the quotient between the number of reads producing a hit with an e-value smaller than 1.0E-40 (using the complete CDS sequences as queries in BLASTN searches with a maximum of 5000 results) and the size in Gb of the SR experiment. Values higher than 100 are in blue, and higher than 10 in green. Values in grey could not be completed.

	Experiment	Tissue	Developmental _stage	sex	*defensin_g2*	*defensin_g3*	*defensin_g7*	*defensin_g9*	*defensin_g11*	*defensin_g13*	*defensin_g15*	*termicin_g1*	*drosomycin_g1*	*drosomycin_g5*	*drosomycin_g6*	*drosomycin_g11*	*drosomycin_g12*	*attacin-like_g1*	*attacin-like_g2*	*blattellicin_g1*	*blattellicin_g4*	SRX Bases
**1**	SRX2892910	Whole body	Embryo day 0		0	0	0	0	0	0	0	0	0	0.5	0	0	0	0	0.4	0	0	8E+09
**2**	SRX2892909	Whole body	Embryo day 0		0	0	0	0	0	0	0	0	0	0.7	0	0	0	0	0	0	0	3E+09
**3**	SRX2892912	Whole body	Embryo day 1		0	0	0	0	0	0	0	0	0	0	0	0	0	0	0	0	0	6E+09
**4**	SRX2892911	Whole body	Embryo day 1		0	0	0.7	0	0	0	0	0	0	6.6	0	0	0	0	0	0	0	2E+09
**5**	SRX2892914	Whole body	Embryo day 2		0	0	0	0	0	0	0	0	0	0	0	0	0	0	0	0	0	1E+10
**6**	SRX2892913	Whole body	Embryo day 2		0	0	0.8	0	0	0	0	0	0	0	0	0	0	0	0	0	0	5E+09
**7**	SRX2892916	Whole body	Embryo day 6		0	0	0.2	0.3	0	0	0	0.3	20.4	761.7	0	0.2	0.9	0	0	0	0	7E+09
**8**	SRX2892915	Whole body	Embryo day 6		0	0	0	0	0.2	0	0	0.4	33.4	959.2	0	0	0	0	0	0	0	5E+09
**9**	SRX2892917	Whole body	Embryo day 13		0.3	0	0	20.8	4.6	2.1	0.3	1	0.3	91.8	1	0	0	0	0	0	0	6E+09
**10**	SRX2892918	Whole body	Embryo day 13		0	0	0.4	12.3	3.9	1.6	0	1	0.6	178.9	1.2	0	0	0	0	0	0	5E+09
**11**	SRX2892920	Whole body	Nymph 1		0.5	1.6	6.2	15.8	1	0	214.2	5.4	1.6	31.8	0.3	0	0	0	0	3.1	3.1	4E+09
**12**	SRX2892919	Whole body	Nymph 1		0.8	0	7	14.5	0.5	0.8	231.4	6.5	5.7	53.8	1.6	0	0.3	0	0.5	0	0	4E+09
**13**	SRX2892922	Whole body	Nymph 3		0	2.6	0	253	2.9	0.7	77.3	0.9	0.4	181.4	11.4	251.7	703	0	0	0.7	0.7	5E+09
**14**	SRX2892921	Whole body	Nymph 3		0	7.8	0.5	149.6	4.4	1.2	76.7	6.5	12.7	220.8	8.8	192	498.7	0	0	0	0	4E+09
**15**	SRX3744245	Whole body	Nymph 5		4.8	3.3	10.8	165.8	3.9	0	95.9	6.9	3.7	184.4	7.8	70.7	152.5	0	0	0	0	5E+09
**16**	SRX3744244	Whole body	Nymph 5		1.8	4.3	4.9	136.8	4.9	0.3	55	0.6	8.6	158.6	5.8	15.7	97.2	0	0	0	0	3E+09
**17**	SRX2746606	Whole body	Nymph 5	female	1.8	6.6	7	180.7	7	0.4	71	0.9	12.3	175.9	7.5	18	115.3	0	0	0	0	2E+09
**18**	SRX2746605	Whole body	Nymph 5	female	5.4	4.2	14.1	206	5.4	0	113.8	8.4	5.1	200.6	7.8	75.4	164	0	0	0	0	3E+09
**19**	SRX3744246	Whole body	Nymph 6		1.3	7.9	6.2	140.6	4.8	0.9	31.8	4.8	13.6	232	3.3	15.3	77.2	0	0	0.7	0.7	5E+09
**20**	SRX3744247	Whole body	Nymph 6		3	21.6	8	140.7	6.9	0.9	44.1	6	15.6	286.4	5.3	27.5	121.6	0	0.5	0.9	1.1	4E+09
**21**	SRX023505	Whole body	Nymph 6		0	0	0	0	0	0	0	0	0	0	0	0	0	0	0	0	0	4E+08
**22**	SRX2746604	Whole body	Nymph 6	female	1.8	10.6	8	171.2	5.8	1.3	36.2	5.8	17.6	240.8	3.8	15.6	86.7	0	0	1	1	4E+09
**23**	SRX2746603	Whole body	Nymph 6	female	4.1	28.6	10.5	178.4	9.2	1	54.6	7.3	19.1	304.2	5.4	31.4	137.2	0	0.6	1.3	1.3	3E+09
**24**	SRX3744248	Whole body	Adult	female	62.5	52.5	13.5	75.4	4.5	0.8	93	13.5	42.3	388.2	1.4	0	0	36.6	5.7	388.2	968.9	5E+09
**25**	SRX3744249	Whole body	Adult	female	72.2	39.8	20.1	47.7	1.3	0.4	72.7	11.5	28.1	209.4	0.4	0	0	40.6	3.6	654.2	711.4	5E+09
**26**	SRX2746608	Whole body	Adult	female	70.3	69.8	17.9	94.1	6.2	1.1	115.5	17.7	54.3	400.6	1.1	0	0	45.2	7.2	1146.3	1204.6	4E+09
**27**	SRX2746607	Whole body	Adult	female	90.8	54.1	27.5	63	1.8	0.6	93.8	13.7	37.6	227.7	0.3	0	0	53.2	4.8	854.5	928.6	3E+09
**28**	SRX884513	Whole body	Adult	female	21.7	0	1.2	91.9	1.6	0	0.8	0	388.6	20.2	0	0	0	20.2	37.6	952.6	1939.3	3E+09
**29**	SRX5916162	Whole body		pooled males and females	3.8	6.5	0.3	0.6	0	0	152.2	0	7.2	0	0	0.1	2.3	0	0	0	0	1E+10
**30**	SRX5916163	Whole body		pooled males and females	2.9	8.2	0	0.8	0.1	0.1	218.9	0	7.3	0	0	1.2	6.4	0	0	0	0	9E+09
**31**	SRX2746602	Whole body	Mixed	pooled male and female	0.1	0	0.8	>14.5	1.4	0.4	>14.5	1.2	6.1	>14.5	1.2	22.5	>14.5	0	0.1	0.2	0.2	7E+10
**32**	SRX551090	Whole body	Mixed	pooled male and female	15.7	5.2	0	41.9	0	0	47.2	2.6	26.2	246.4	0	15.7	34.1	21	36.7	34.1	39.3	4E+08
**33**	SRX548460	Whole body	Mixed	pooled male and female	61.6	5.9	2.9	214.1	14.7	0	32.3	0	5.9	648.3	2.9	0	0	134.9	240.5	20.5	17.6	3E+08

	Experiment	Tissue	Developmental_stage	sex	*defensin_g2*	*defensin_g3*	*defensin_g7*	*defensin_g9*	*defensin_g11*	*defensin_g13*	*defensin_g15*	*termicin_g1*	*drosomycin_g1*	*drosomycin_g5*	*drosomycin_g6*	*drosomycin_g11*	*drosomycin_g12*	*attacin-like_g1*	*attacin-like_g2*	*blattellicin_g1*	*blattellicin_g4*	SRX Bases
**34**	SRX2892907	Eggs	Non-fecundated eggs		0	0	0	16.6	0.9	0	0	0	1.8	49.8	0.3	0	0	0	0	0.2	0.2	9E+09
**35**	SRX2892908	Eggs	Non-fecundated eggs		0.3	0	0	13.1	0.3	0	0	0	2.2	24.7	0	0	0	0	0	0	0	8E+09
**36**	SRX798229	Epidermis\; including cuticle layers	Nymph 6		0	0	0	25.8	0	0	0	0	25.8	8.6	0	0	0	0	0	0	0	1E+08
**37**	SRX798230	Fat body	Adult	female	0	0	0	0	0	0	0	0	0	12	0	0	0	0	0	0	0	8E+07
**38**	SRX475282	fat body\; ovary\; epidermis	Adult	female	0	0	0	59.5	0	0	0	0	0	19.8	0	0	0	0	0	0	0	5E+07
**39**	SRX475279	fat body\; ovary\; epidermis	Adult	female	0	0	0	25.5	0	0	0	0	0	25.5	0	0	0	0	0	0	0	4E+07
**40**	SRX475028	fat body\; ovary\; epidermis	Adult	female	0	0	0	0	0	0	0	0	0	25.8	0	0	0	0	0	0	0	4E+07
**41**	SRX3189901	Head	Adult	male	5.3	0	11.1	11.1	5.4	0.5	0.4	0.1	0.6	13.4	0.6	0	0	0.9	4.4	0	0	2E+10
**42**	SRX3189902	Head	Adult	male	9.2	0	28.1	17.6	4.4	0.3	0	3.1	1.1	17.8	0	0	0	8.5	26.7	0	0	1E+10
**43**	SRX682022	Head	Adult	not determined	0	0	0	0	0	0	0	0	0	0	0	0	0	0	0	0	0	1E+10
**44**	SRX796238	Ovaries	Adult	female	0	0	0	0	0	0	0	0	0	68.5	0	0	0	0	0	0	0	7E+07
**45**	SRX796239	Ovaries	Adult	female	0	0	0	0	0	0	0	0	0	44.3	0	0	0	0	0	0	0	5E+07
**46**	SRX023504	ovary	Adult	female	0	0	0	0	0	0	0	0	0	0	0	0	0	0	0	0	0	3E+08
**47**	SRX798228	Ovary	Adult	female	0	0	0	10.9	0	0	0	0	0	43.6	0	0	0	0	0	0	0	9E+07
**48**	SRX796244	Ovaries	Nymph 6	female	0	0	0	0	0	0	0	0	0	0	0	0	0	0	0	0	0	4E+07
**49**	SRX6380685	ovaries\; colleterial gland\; and fat body	Adult	female	0	0	0	0	2.3	0	0	0	0	5.3	0	0	0	0	0	0	0	9E+09
**50**	SRX801770	Tergites 7-8	Nymph 6		0	0	0	0	0	0	0	0	0	15.3	0	0	0	0	0	0	0	7E+07
**51**	SRX801771	Tergites 7-8	Nymph 5		0	0	19.3	0	0	0	0	0	0	19.3	0	0	0	0	0	0	0	5E+07
**52**	SRX801772	Tergites 7-8	Nymph 6		0	0	968.1	20.2	0	0	0	0	0	40.3	0	0	0	0	0	0	0	5E+07
**53**	SRX801773	Tergites 7-8	Nymph 6		0	0	24.2	24.2	0	0	0	0	0	48.4	0	0	0	0	0	0	0	4E+07
**54**	SRX6380684	testes\; accessory gland\; and fat body	Adult	male	0	0	0	0	0	0	0	0	0	0	0	0	0	0	0	0	0	3E+10
**55**	SRX790658	Wings	Nymph 5	male	0	0	18.4	0	0	0	0	0	0	18.4	0	0	0	0	0	0	0	5E+07
